# A scoping review of models of care and services for nausea and vomiting in pregnancy and hyperemesis gravidarum

**DOI:** 10.1186/s12884-025-08093-y

**Published:** 2025-10-03

**Authors:** Pippy Walker, Alexis Turner, Swathi Sridharan, Xinrui Dong, Carmen Huckel Schneider, Andrew Wilson, Amanda Rush

**Affiliations:** 1 Leeder Centre for Health Policy, Economics & Data, Sydney School of Public Health, Faculty of Medicine and Health, The University of Sydney, Sydney, NSW Australia; 2 Sydney School of Public Health, Faculty of Medicine and Health, The University of Sydney, Sydney, NSW Australia

**Keywords:** Pregnancy, Hyperemesis gravidarum, Morning sickness, Models of care, Health services

## Abstract

**Background:**

Nausea and vomiting in pregnancy and hyperemesis gravidarum have significant physical and psychological impacts on pregnant women. Women are often unable to participate in everyday care and work activities, potentially affecting individual wellbeing and ability to contribute to the economy. Research priorities for hyperemesis gravidarum have been established, however little is known about the current landscape of literature on the health and social care provided to affected women. To inform decision makers on effective models of care and identify remaining evidence gaps, this scoping review aimed to map existing literature that evaluated health and social care delivery arrangements for pregnant women with nausea and vomiting and hyperemesis gravidarum.

**Methods:**

Seven databases (Medline, EMBASE, CINAHL, PsycInfo, Maternity and Infant Care, Cochrane Library and Scopus) were searched in August 2023 and again in March 2024 to identify evaluations of health care delivery arrangements for nausea and vomiting in pregnancy and hyperemesis gravidarum. Database searches were supplemented by a Google search for additional peer-reviewed and grey literature. Data from included publications were extracted and synthesised to report on models of care identified, countries, study designs, outcome measures and key findings.

**Results:**

Seventy-two records describing 60 separate models of care were identified. Models of care were grouped into hospital settings (*n* = 34 records), digital health (*n* = 19 records), group-based care (*n* = 11 records), and primary care (*n* = 8 records). Literature in the hospital setting addressed outpatient management (*n* = 15 records), inpatient management (*n* = 5 records), comparisons of outpatient and inpatient management (*n* = 10 records), in-home care (*n* = 3 records) and emergency departments (*n* = 1 record). Digital health care evaluations included telephone and web-based support (*n* = 14 records) and mobile phone applications (*n* = 5 records).

**Conclusions:**

This scoping review has identified the need for further research on approaches to care provision for nausea and vomiting in pregnancy and hyperemesis gravidarum. Whilst some positive findings on patient outcomes, patient experiences and reduced hospital stays resulting from outpatient hospital management pathways were reported, much of this research was observational and limited to UK settings. Other research gaps identified pertained to the effectiveness of primary care, social support and in-home care services, including economic evaluations.

**Supplementary Information:**

The online version contains supplementary material available at 10.1186/s12884-025-08093-y.

## Background

Hyperemesis Gravidarum (HG) is a severe form of nausea and vomiting in pregnancy (NVP), and the most common cause of hospitalisation during the first and second trimesters [1]. Evidence suggests that while 70% of pregnant women experience nausea and vomiting, the prevalence of HG is estimated to be much lower (between 0.3%−3.6% globally) [2]. Characterised by early onset of symptoms, severe vomiting and/or nausea, and an inability to eat and/or drink normally or participate in daily living activities [3], HG has significant effects on the physical and mental health of pregnant women [4]. Unmanaged cases of HG can be life threatening, with severe complications including Wernicke’s encephalopathy, electrolyte imbalances and vitamin K deficiency [5].

Whilst recent advances have been made in better understanding the cause of HG and in developing curative treatments [6], such treatments are not yet available. Current management options for women with severe NVP and HG include pharmaceutical interventions, intravenous rehydration, outpatient care and other supportive treatments such as acupuncture and acupressure [7].

To support the implementation of evidence into practice, several clinical practice guidelines are available to inform health care providers and health policy and practice stakeholders responsible for service delivery models and ongoing quality improvement. A recent systematic review and appraisal of these guidelines found that national guidelines published in Australia, the UK, the USA and Canada provided similar recommendations [8], with UK guidelines updated since publishing of the review [9].

Despite the availability of relatively harmonised guidelines to support practice, inefficiencies remain in service delivery models for women with NVP and HG. In some cases, there are no or few health and social care options available. In the USA, high and increasing rates of presentation to the emergency department for HG have been reported [10, 11]. This has resulted in increased emergency department costs, with one study citing a 65% increase in patient costs [10]. Similarly in the UK, hospital admissions for HG are increasing. This has resulted in calls for further investigation into the role of improved primary care management [12].

Research in Australia suggests that there may be a lack of adequate NVP and HG services, with less than half of women reportedly accessing health care support for NVP [13, 14]. Women’s dissatisfaction with the care provided by their GPs has been reported in Norway, with a need for greater awareness and knowledge among health care professionals recommended [15]. Qualitative research in the Netherlands identified several suggestions from women with HG to improve care based on their experiences [16]. These included early access to care and medical intervention, more uniform information and policies regarding HG treatment, the option for in-home treatment, and psychological support [16].

The international patient-clinician priority setting initiative James Lind Alliance established research priorities for HG in 2021, which included whether treatment guidelines improve management and outcomes, how they can be implemented, barriers to accessing services and how these can be overcome, and what health services exist and how they are organised [17]. A separate comprehensive systematic review has been conducted to address the top 10 priority research questions for HG, however this did not include these priorities [18]. The aim of this scoping review was to map the evidence related to health and social care delivery arrangements for women with NVP or HG, to inform decision makers on current effective models of care (MoC) and identify any remaining evidence gaps.

## Methods

The conduct of this systematic scoping review was informed by Arksey and O’Malley’s (2005) methodological framework [19]. It follows the PRISMA extension for scoping reviews (PRISMA-ScR) for reporting [20] (see Additional File 1 for completed PRISMA-ScR checklist).

### Identifying the research question

The parameters of the review were discussed by the research team in mid-2023 and a protocol for the conduct of the review was established at this time. Literature that was related to improving the ways in which women accessed or received information, support and care for NVP or HG was identified as being of interest. Participants of interest were pregnant women with nausea and vomiting or HG that accessed care across any health or social care sector. The key concept of the review was to identify initiatives that focused on improving the way that health or social care was delivered, including both existing and new MoC and services.

### Identifying relevant studies

A comprehensive literature search strategy was developed in consultation with medicine and health academic liaison librarians, which included both peer-reviewed and grey literature. Medline, EMBASE, CINAHL, PsycInfo, Maternity and Infant Care, Cochrane Library and Scopus were searched using key terms related to NVP and HG, and MoC, for articles published from January 2012. An initial search was conducted on 8 August 2023, with an updated search conducted on 19 March 2024 to identify additional publications.

Using similar search terms, a Google search using a private browser and clear cache in Microsoft Edge was conducted to identify relevant websites from Australia, New Zealand, United States of America, United Kingdom and Canada. These countries were selected as representative of health systems from OECD countries, with similar percentages of GDP expended on health care [21]. Each search string was captured with both government and organisation domain Names where available, with their respective country domain. Depending on the number of results, up to 50 search results were saved for each country and domain. Searches were conducted from 21 to the 29 of February 2024 (see Additional File 2 for full search strategy).

Reference lists of included articles were hand searched, and experts were consulted for the identification of any additional literature suitable for inclusion. Twenty-four experts within our health service networks were contacted via email in February 2024, to which four responded with literature to consider. Respondents included an obstetrician-gynaecologist, an obstetric physician, a telephone service counsellor for pregnant women, and a clinical midwife consultant.

### Study selection

All records published from 2012 in English were considered for this review, with no limitations placed on the country or type of publication. The establishment of inclusion criteria was an iterative process, which has been recognised as necessary for scoping reviews [22]. Literature screening was initially guided by the study aim and a set of screening questions (see Additional File 2). A conservative approach to the inclusion of records was applied in order for researchers (PW, AR, AT) to discuss and reach consensus on relevance for the review, and to define the scope of literature to be included.

As the primary focus of the review was to inform health service decision makers (i.e. those with influence and authority on how care is designed to meet consumer needs, by whom care is provided, where care is provided and with what supports care is provided), literature that evaluated (or planned to evaluate) initiatives designed to improve health and social care delivery arrangements for women with NVP and/or HG were included.

Delivery arrangements encompass MoC or services, including group-based services; packages of care and care pathways; information to support patient self-management; health care infrastructure such as where care is provided, facilities, integration of services; and supports for care provision (e.g., electronic health records, other information and communication technology, quality monitoring and improvements systems) [23, 24]. Delivery arrangements also include the health workforce, including considerations for supply, distribution and continuity of care [23, 24]. Literature concerning the clinical management of NVP or HG, such as trials evaluating the efficacy of medications or other clinical treatments were not included in this review, unless they also involved an innovative or novel approach to care delivery (e.g., group-based care, telephone or telehealth consultations).

The review focused on literature that tested or implemented potential health system solutions to improve health and social care. It did not include literature that aimed to clarify health system issues by assessing the status quo (e.g. rates of presentations to the emergency department, antiemetic prescribing rates, women’s experiences with standard care, knowledge and confidence of health care professionals). To maintain focus on the way that health and social care for NVP and HG is delivered, the review did not include health care professional training programs or the development of patient resources.

Peer-reviewed records identified during the search phase were imported into Covidence [25] and duplicates were removed. One author (PW) screened titles and abstracts for inclusion, of which 10% of records were reviewed by a second author (AR). Any disagreements were discussed and resolved. The same approach was taken for full text review, with both authors (PW, AR) checking all full text articles for inclusion. Reference lists of literature reviews were screened for any additional records. Grey literature sources were exported into Microsoft Excel and were screened by one author (AT), with a 10% sample reviewed by another author (PW) and disagreements discussed and resolved. Three authors (AT, PW, AR) checked and agreed upon the final inclusion of grey literature sources.

### Charting the data

A data charting template was developed by the research team and updated over the course of the data extraction process (see Additional File 3). Data items included the identified reference, country, health care setting, type and description of the initiative or MoC, patient cohort (NVP, HG, both), type of evaluation, outcomes and outcome measures (including the use of validated patient reported outcome measures (PROMs) and patient reported experience measures (PREMs)), key findings, study limitations and any other relevant notes. In line with the broad focus of scoping reviews to identify all literature available on the topic of interest, a critical appraisal of included records was not conducted [20]. Data extraction of included records was split between four researchers (PW, XD, SS, AT), with two authors (PW, AR) cross-checking all records for accuracy and completeness.

### Collating, summarising and reporting results

Records were grouped in a data extraction table according to health care sector or type of delivery arrangement. Subheadings were established and the presentation of data was streamlined to ensure a consistent approach to reporting. One author (PW) synthesised the data, in consultation with the research team. Results were written using a narrative synthesis, supplemented with tables for key findings.

## Results

### Overview of included literature

**Fig. 1:**
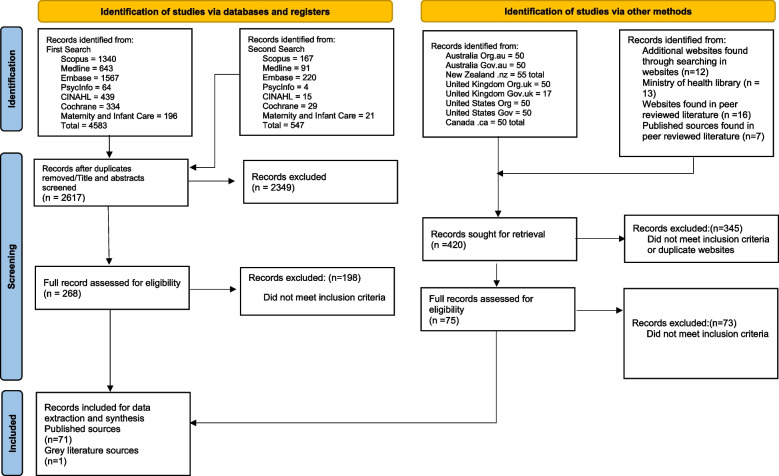
PRISMA Diagram. From Page et al. 2021 [26]

A total of 72 records suitable for inclusion were identified from a total of 3037 screened peer-reviewed and grey literature sources (Fig. 1). From the 72 included records, 60 separate MoC were identified. The included literature is summarised in Tables 1 and 2.

**Table 1:** Summary of included literature (*n* = 72 records)

		**N (%)**
**Countries**	UK	28 (38.9%)
	Iran	12 (16.7%)
	Norway	7 (9.7%)
	USA	5 (6.9%)
	Ireland	4 (5.6%)
	Australia	2 (2.8%)
	India	2 (2.8%)
	China	2 (2.8%)
	Canada	2 (2.8%)
	Finland	1 (1.4%)
	Denmark	1 (1.4%)
	Turkey	1 (1.4%)
	Taiwan	1 (1.4%)
	Egypt	1 (1.4%)
	Japan	1 (1.4%)
	Pakistan	1 (1.4%)
	New Zealand	1 (1.4%)
**Year of publication**	2012—2014	18 (25%)
	2015—2017	19 (26.4%)
	2018—2020	16 (22.2%)
	2021—2024	19 (26.4%)
**Type of publication**	Full text primary study	32 (44.4%)
	Study abstract	26 (36.1%)
	Trial registration	9 (12.5%)
	Study protocol (including protocol abstracts)	4 (5.6%)
	Website	1 (1.4%)

**Table 2:** Summary of included models of care (*n* = 60)

		**N (%)**
**Study design**	Observational	23 (38.3%)
	Randomised Controlled Trial	18 (30.0%)
	Quality Improvement	13 (21.7%)
	Quasi experimental	5 (8.3%)
	Cost Utility Analysis	1 (1.7%)
**Population**	NVP	24 (40.0%)
	HG	30 (50.0%)
	NVP and HG	6 (10.0%)
**Patient Reported Outcome Measures (PROMs)**	Pregnancy-Unique Quantification of Emesis and Nausea (TOTAL):	23 (38.3%)
	• PUQE-12 [27]	4 (6.7%)
	• PUQE-24 [28]	7 (11.7%)
	• Modified PUQE [29]	4 (6.7%)
	• PUQE version not specified	8 (13.3%)
	Nausea and Vomiting of Pregnancy specific HRQoL (NVPQoL)	5 (8.3%)
	Rhodes Index of Nausea, Vomiting and Retching (INVR)	5 (8.3%)
	36-Item Short Form Survey (SF-36)	2 (3.3%)
	12-item Short Form Survey (SF-12)	2 (3.3%)
	EQ5D (HRQoL)	2 (3.3%)
	Wellbeing rating/score	2 (3.3%)
	Quality of Life (not specified)	2 (3.3%)
	Nausea and Vomiting in Pregnancy Instrument (NVPI)	1 (1.7%)
	Pregnancy Symptoms Inventory	1 (1.7%)
	Hyperemesis Level Prediction (HELP) Score	1 (1.7%)
	Hyperemesis Impact of Symptoms Questionnaire [30]	1 (1.7%)
	Pregnancy Related Anxiety Questionnaire (PRAQ-17)	1 (1.7%)
	Quality of Life Scale	1 (1.7%)
	WHO-5 Wellbeing Index	1 (1.7%)
	Depression Anxiety and Stress Scale 21 (DASS-21)	1 (1.7%)
	Cohen’s perceived stress questionnaire	1 (1.7%)
	Hope for life scale	1 (1.7%)
	Edinburgh questionnaire	1 (1.7%)
	Self-care agency (ESCA 35)	1 (1.7%)
	Multidimensional scale of perceived social support (MSPSS)	1 (1.7%)
	Decisional Conflict Scale (DCS)	1 (1.7%)
	Hudson sexual satisfaction questionnaire	1 (1.7%)
	Eating and drinking scoring system	1 (1.7%)
	**Visual Analogue Scales (VAS):**	
	Symptom distress	1 (1.7%)
	Physical Quality of Life	1 (1.7%)
	Mental Quality of Life	1 (1.7%)
	Stress	1 (1.7%)
**Patient Reported Experience Measures (PREMs)**	Study specific questionnaire	5 (8.3%)
	Survey on satisfaction or experience (details not provided)	5 (8.3%)
	Client Satisfaction Questionnaire	2 (3.3%)
	Satisfaction Score [31]	1 (1.7%)
	Patient Satisfaction Questionnaire [32]	1 (1.7%)
**No PROMs or PREMs used**		22 (36.7%)

References have been included for PROM and PREM tools where they were provided or where the authors were able to identify them in relevant database searches

HRQoL health related quality of life

There were 31 hospital MoC identified from 34 records, 15 digital health MoC from 19 records, 8 group-based care MoC from 11 records, and 6 primary care MoC from 8 records. Hospital based MoC included outpatient management (n = 15 MoC from 15 records), inpatient management (n = 4 MoC from 5 records), comparison of outpatient and inpatient management (n = 8 MoC from 10 records), in-home care (n = 3 MoC from 3 records), and emergency departments (n = 1 MoC from 1 record). Digital health included telephone and web-based support (n = 12 MoC from 14 records) and mobile applications (n = 3 MoC from 5 records). Literature in each group is described below, with a summary of hospital MoC provided in Tables 3 and 4 and full details of all data extracted available in Additional File 3.

**Table 3:** Summary of hospital models of care and outcome measures

	**Records**	**Description of model of care/intervention**	**Outcome measures**	**PROMs/PREMs**
**Outpatient management**	Ajufo (2013) [51]	Day admission service for HG	Hospital admissions, length of stay	Nil
	Buchanan, Hendry and Currie (2016) [52]	Introduction of a flow chart for practitioners, new maternity assessment unit guideline, assessment sheet and GP communication sheet	Hospital admissions, assessment sheet completion rates, compliance with guideline protocols	Nil
	Channing and Doraiswamy (2021) [53]	New day case hyperemesis unit for rapid rehydration and antiemetics, staff education	Proportion managed in new unit	Nil
	Clark (2016) [54]	Outpatient hyperemesis service	Hospital length of stay	Nil
	Cobb et al (2014) [55]	Alteration of operating times of hydration unit and staff education	Hospital admissions and patient level data	Nil
	Coleman et al (2014) [37]	HG day case service	Hospital admissions, protocol compliance audit, patient satisfaction	PREMs-NS
	Doherty et al (2023) [33]	Multidisciplinary HG day case service - IRIS (Intravenous fluids, Rest, Insight and Support) hydration clinic	Women’s experiences (interviews)	Nil
	Fernandopulle and Doraiswamy (2021) [56]	New day case pathway for severe NVP and ‘hyperemesis pack’ to support prescribing and provide patient information	Hospital admissions and patient level data	PUQE (used clinically, not for evaluation)
	Ijaz et al (2016) [57]	New rapid hydration clinic using fast hydration protocol	Hospital admissions, patient level data (e.g., demographics, gestation, health status and indication for treatment)	Nil
	Khan, Karpate and Shehmar (2013) [58]	Hyperemesis day centre using a nurse run protocol for rapid hydration	Hospital admissions, protocol adherence, patient experiences	PREMs-NS
	O’Brien et al (2023) [35]	Multidisciplinary HG day case service - IRIS (Intravenous fluids, Rest, Insight and Support) hydration clinic	NVP symptoms, well-being, quality of life, gestational weight gain, dietary intakes, infant birthweight, women’s experiences (interviews)	PUQE-24, PSI, SF-12, WHO-5
	Pilling, Knowles and Khalil (2018) [59]	New ambulatory care pathway for NVP, staff education and training, updated guidelines, patient proforma	Hospital admissions	Nil
	Tompsett et al (2013) [60]	Ambulatory care bundle including IV fluid hydration, patient information, and discharge with cyclizine, thiamine and folic acid	Hospital admissions	PUQE (used clinically, not for evaluation)
	Ucyigit (2020) [34]	Ambulatory hyperemesis unit run by nursing staff	Hospital admissions	Nil
	Wang and Ma (2018) [36]	Ambulatory hyperemesis gravidarum initiative focused on devising improvement strategies based on clinical guidelines compliance audits and patient feedback	Hospital admissions and patient experiences	PREMs-NS
**Inpatient**	Fletcher et al (2013 and 2015) [38, 39]	Patient assessment using Hyperemesis Impact of Symptoms Questionnaire (HISQ), individualised care plan and advice to support symptom management after discharge, follow up phone call.	Social functioning, satisfaction, hospital admissions, length of stay, health status, NVP symptoms, economic outcomes	HISQ, Short Form Health Survey (SF-36), EQ5D, PUQE-12, Client Satisfaction Questionnaire (CSQ)
	Laitinen et al (2022) [42]	Testing the usability of the Finnish-translated PUQE in hospitalised women with HG	Usability of Finnish PUQE	PUQE-12, VAS
	Lloyd, Ramskill and Sharma (2014) [40]	Proforma for practitioners to prompt investigations, medications, and fluid resuscitation for patients with HG	Fluid resuscitation practices, prescribing practices, hospital length of stay and practitioner feedback	Nil
	Nam, Patil and Sieunarine (2018) [41]	New NVP/HG management protocol including rapid rehydration, anti-emetic administration, and thiamine prescription	Hospital length of stay, practice audit on proforma completion	Nil
**Home**	Canty et al (2022) [61]	Hospital in The Home (HiTH) – Intravenous hydration treatment at home over 3 days	Hospital admissions, costs	PUQE, QoL, satisfaction survey
	Carlon and Kiroglu (2022) [62]	Medical Obstetrics @ Home (MoaH) – Integrated model for complex obstetric conditions at home with ongoing care via telehealth	NS	Nil
	Ostenfeld et al (2023) [63]	Home intravenous fluids from gynaecology department, development of a patient manual	Patient satisfaction	Study PREMs survey
**ED**	Skalley et al (2018) [64]	New proforma for HG care in ED	Patient management indicators, discharge outcomes, admissions, length of stay	Nil

*PROMs/PREMs* Patient reported outcome/experience measures, *NVP* Nausea and Vomiting in Pregnancy, *HG* Hyperemesis gravidarum, *NS* Not Specified, *PUQE* Pregnancy Unique Quantification of Emesis, *PSI* Pregnancy Symptoms Inventory, *SF-12* 12 item short form health survey, *WHO-5* Wellbeing index, *VAS* Visual Analogue Scale

**Table 4:** Summary of literature comparing inpatient and outpatient management of NVP and HG

**Records**	**Description of comparison to IPM**	**Outcome measures**	**PROMs/PREMs**	**Key Findings**
Dean and Marsden (2017) [48]	HG Day Units (HGDU) which provide rapid IV rehydration and medication	Patient satisfaction	Satisfaction questionnaire	Satisfaction was greater among OPM group. OPM group had fewer days in hospital
Dean and Marsden (2017) [65]	HGDU in early pregnancy units where women receive rapid IV rehydration and medication	Reasons for patient satisfaction and dissatisfaction of OPM and IPM	Satisfaction questionnaire	Highlighted need for staff education and patient information, with OPM particularly preferred by women with children at home
Hordern et al (2013) [66]	Outpatient management involving IV rehydration and anti-emetics	Hospital length of stay, readmission rates, medications prescribed and ketones	Nil	Similar readmission rates between groups. Reduced length of stay if re-admitted for OPM group
McCarthy et al (2014) [47]	Day care treatment for NVP	Hospital length of stay, quality of life, volume of IV fluids, antiemetics and multivitamins administered, patient satisfaction	EQ5D, Client satisfaction questionnaire	Day care reduces hospital inpatient stay and was satisfactory to patients
McParlin et al (2016) [46]	Midwifery-led day management of severe NVP involving rapid rehydration, symptom relief and telephone support	NVP symptoms. quality of life, satisfaction with care, birth outcomes, readmission rates and hospital length of stay	PUQE-12, SF-36, Satisfaction score	Equal effect on NVP symptoms, quality of life and satisfaction, and reduced hospital length of stay for OPM
Mitchell-Jones et al (2017) [43–45]	Ambulatory care involving daily attendance to an outpatient unit with inpatient care for severe NVP	NVP symptoms, duration of treatment, improvement in symptom scores and ketonuria, reattendances	PUQE-12, Wellbeing rating, Eating and drinking scoring system	Equal effect on PUQE score at 48 hours, improvements in symptoms scores, ketonuria, duration of treatment and reattendance rates
Murphy et al (2016) [50]	Day care management of NVP involving IV fluids and antiemetics	Total costs and QALYs for day care and inpatient management of NVP	QALYs derived from EQ5D and SF-36 scores	OPM is less costly and more effective than IPM
Ryan et al (2019) [49]	Day case management involving referral to an on-call physician, initial investigations and treatment	Patient satisfaction, hospital length of stay, and biochemical parameters.	Patient satisfaction questionnaire	Higher satisfaction and shorter length of stay in OPM

*PROMs/PREMs* Patient reported outcome/experience measures, *NVP* Nausea and Vomiting in Pregnancy, *HG* Hyperemesis gravidarum, *IV* Intravenous, *PUQE* Pregnancy Unique Quantification of Emesis, *SF-36* 36 Item short form health survey, *QALY* Quality Adjusted Life Year, *OPM* Outpatient management, *IPM* Inpatient management

### Hospital models of care

#### Outpatient management

All literature identified on outpatient management of NVP or HG was based in the UK (n = 13 records) or Ireland (n = 2 records). Two records were full text studies [33, 34], with the remainder conference abstracts or study protocols. Observational studies, usually involving an audit as part of a quality improvement activity were predominant, with four studies involving an additional qualitative component to explore women’s experiences of care [33, 35–37]. Most observational studies reported short follow up periods, however one retrospective audit of an ambulatory hyperemesis unit reviewed impact on inpatient admission rate and length of stay over 12 years of operation [34]. This study estimated a cost saving of £100,000 per year from reduced length of stays [34].

#### Inpatient management

Two of five studies focusing on improving inpatient management of NVP and/or HG related to the same UK randomised controlled trial (RCT) [38, 39], two were UK quality improvement projects [40, 41], and one was a prospective cohort study in Finland [42]. The interventions involved holistic assessment and tailored management plans for patients based on the Hyperemesis Impact on Symptoms Questionnaire [38, 39], development and implementation of new protocols to improve quality of care and reduce length of admission [40, 41], and an assessment of the usability of the pregnancy-unique quantification of emesis (PUQE) translated into Finnish for women hospitalised with HG [42]. The UK RCT also incorporated a cost-utility analysis to evaluate the impact of a tailored plan of care to address women’s individual needs. It did not find any significant improvement in quality of life or any cost savings [38, 39].

#### Comparison of outpatient and inpatient management

Included studies comparing care in outpatient and inpatient settings were conducted in the UK (*n* = 8), and Ireland (*n* = 2). RCTs found that ambulatory management was as effective as inpatient care at improving symptoms [43–46] and quality of life [46]; and outpatient management resulted in reduced time in hospital [46, 47]. Several studies also reported either equal or greater satisfaction of care associated with outpatient management compared to inpatient care [46–49] (see Table 4). A cost-utility analysis found that day care management was less costly and more effective compared with inpatient management of NVP [50].

#### In-home care

Literature on in-home care found for HG was limited to two Australian protocol abstracts [61, 62] and one quality improvement project in Denmark [63]. Protocols described the implementation of a hospital in the home (HiTH) model and a medical obstetrics at home (MoaH) model, with only the HiTH model including details of a planned evaluation [61]. The Danish quality improvement study [63] reported on the introduction of home treatment as an aspect of a new MoC where intravenous fluids were provided in the gynaecology department. While two recipients of in-home care reported high satisfaction, both experienced complications associated with their existing central venous catheters. The treatment team concluded that these should only be offered as a last resort and that treatment at home for patients with midlines could also be considered [63].

#### Emergency department

One quality improvement project with practice audits was identified in an emergency department setting [64]. The project involved the collaborative development of a proforma for a new clinical pathway by emergency department and obstetrics and gynaecology teams in the UK, with the aim of reducing admission rates to obstetrics and gynaecology departments through improving emergency quality of care. Audits identified an improvement in compliance with prescribing guidelines for HG in emergency, and a reduction in admission rates to obstetrics and gynaecology [64].

### Digital health

#### Telephone and web-based support

The most frequently reported service in the identified literature was the Motherisk NVP Helpline (*n* = 5) [67–71]. Established in Canada in 1995, and also available in the USA, the Motherisk NVP helpline was the first service established to counsel expecting and planning mothers on the management of NVP [68]. Within the service, there is little evaluation data presented, with three descriptive studies reporting on numbers of calls, geographic locations of callers, and reasons for calls in Canada [67] and advice and prescriptions provided in the USA [68–70]. A fourth study compared callers with HG to callers with NVP to understand any differences in demographics, clinical presentations and clinical outcomes of mothers and babies [71]. Women with HG required and sought more counselling compared to women with NVP, with the abstract concluding that service provision was associated with more favourable outcomes for women with HG [71].

Descriptive studies reporting on caller characteristics and reasons for calling other telephone helplines were also identified, including the Garbha-Swasthya helpline in India [72] and the University of North Carolina Health Care System Drug Information Centre in the USA [73]. The Indian service is specific for pregnant women and their health care professionals, whereas the USA service is a general drug information centre for health care professionals. The USA service reported that obstetric calls (including those in relation to HG) had increased over time [73].

Trials conducted in Iran [74–76], Turkey [77] and Taiwan [78] have evaluated the effectiveness of telephone-based counselling and support for women with NVP. A research study in Iran recruited 60 women with NVP in their first trimester (n = 30 each in intervention and control groups), with the intervention group receiving social support via phone twice per week for four weeks with each call lasting 15–20 minutes. One associated publication reported significant improvements in NVP (measured via modified PUQE) [75], with another reporting improvements in perceived social support [76].

Similarly, a quasi-experimental trial involving weekly nurse telephone counselling in Turkey found that NVP symptoms resolved earlier in the intervention group compared to the control, however this difference was only significant for women with mild to moderate NVP [77]. Provision of individualised health education and supportive phone calls for 40 women with NVP compared with 39 women receiving routine nursing care in Taiwan also found that the intervention resulted in significantly lower severity of nausea and vomiting and perceived level of symptom distress, and significant improvements in quality of life [78].

In addition to telephone-based support, descriptive studies observing the use of web-based services including the SafeMotherMedicine service in Norway for pregnant and breastfeeding women [79] and the e-Sanjeevani online platform to provide obstetrics and gynaecology specialist support to providers and their patients in India [80] were identified. The mode of teleconsultation using the e-Sanjeevani platform varied between text-based interaction and real-time video, audio and text interactions, with 7% of inquiries related to NVP or HG [80].

#### Mobile applications

Five records (2 peer-reviewed publications, 1 abstract, 1 trial registration and 1 website) relating to three different mobile applications (‘apps’) or use of mobile phones to deliver care to women with NVP were identified, with one each from Norway, the USA and the UK. One app [81] has been designed to track symptoms daily to support prenatal review appointments, and the other two served to collect data about symptoms and then generate appropriate advice based on symptom severity [82–85].

One RCT in Norway assessed the impact of an app on NVP related symptoms compared with standard maternal care [82, 83], for which no impact was found. Issues reported in this study that may have influenced reported findings included that 15% of women in the intervention group were beyond the first trimester, that there was a 25% drop out rate, that target recruitment was not reached, and that women may have reported their symptoms at different times each day [82, 83]. The other two apps had observational study designs to assess patient and healthcare provider acceptability [81] and feasibility of collecting data [84, 85].

In addition to the above-mentioned studies, evaluation of a community pharmacist consultation in Norway by Truong et al. [86] reported under Primary care (below) also mentioned the development of a patient centered mobile app that had been user tested. The abstract also stated that patient generated data from the app could be linked to information from national health care registries, however no evaluation findings were reported [86].

### Group-based care

Care provided to women in groups for NVP or HG was identified in controlled trials from Iran, Egypt, China and Japan. The predominant MoC was education that was based on the Ottawa nutritional guideline [87], with outcomes of interest including NVP symptoms and quality of life [88–91]. Where results were reported, interventions had had significant positive impacts on quality of life [88, 90]. Studies on other models provided limited information, with a mix of trial registrations, protocols and conference abstracts identified. These interventions involved group counselling based on cognitive behavioural therapy [92], counselling based on the hope therapy approach [93], group biofeedback treatment [94], and a nursing care program based on Miwa’s reflective practice [95]. The delivery of group-based care ranged from 2 to 10 sessions each running for between 30 to 90 min provided over up to 4 weeks (see Additional File 3 for details).

### Primary care

Eight records were identified that focused on four different care delivery arrangements for NVP or HG in the primary care setting. The first of these focused on improving communication between gynaecologists and primary care providers to improve primary care management of HG, and subsequently reduce the rate of referral to specialist care [96]. Gynaecologists in Pakistan provided two awareness sessions over three months to GPs to communicate the use of previously developed standard operating procedures, which resulted in a 31% reduction in HG referrals during the first trimester [96]. The second arrangement involved the evaluation of a community pharmacy consultation for pregnant women in Norway, for which almost half of participants had moderate to severe NVP [86, 97–99].

In a New Zealand urgent care setting, a retrospective quality improvement audit was undertaken to assess waiting periods and treatment times of women presenting with NVP or HG [100]. To address long wait times and other inefficiencies in care, a nursing care clinical pathway was proposed, which offered nurse-led assessment and development of a plan of action [100]. Lastly, two RCTs in Iranian healthcare centres evaluated the effect of counselling on NVP [101, 102]. Counselling sessions were provided by midwives, which resulted in a significant reduction in the severity and frequency of nausea and vomiting compared to routine prenatal care [101].

## Discussion

Mapping the body of literature on MoC and services for NVP and HG demonstrated an overall scarcity, as well as specific gaps. To maximise findings and insights, this scoping review included conference abstracts, study protocols and trial registrations in addition to full text publications. This resulted in a total of 72 records, averaging to six records per year across all health care settings over the 12-year search period. A previous scoping review on literature concerning causes, prevention, cure, consequences and clinical management of HG also highlighted significant gaps and a need for more original research and systematic reviews [18]. Table 5 provides a summary of gaps identified in this literature review, and arising research questions.

**Table 5:** Research questions arising from identified literature gaps

**Identified gap**	**Research Question**	**Benefit**
Limited experimental and quasi-experimental studies, particularly outside of the UK	What are the effects of outpatient hospital management pathways for NVP and HG in experimental or quasi-experimental studies across diverse health settings?	Will help strengthen casual evidence and generalisability beyond the UK
Lack of focus on PROMs and PREMs	How do different care delivery models for NVP and HG impact patient-reported outcomes and experiences?	Prioritises patient-centred care
Underrepresentation of primary care, social support and in-home services	What is the effectiveness of and satisfaction with primary care, social support and in-home care models of care and services for NVP and HG?	Expands understanding beyond hospital settings and supports a biopsychosocial approach to care
Limited dissemination of quality improvement (QI) initiatives	How can partnerships between researchers, hospital departments, and health services facilitate the publication of QI projects related to NVP and HG care?	Addresses publication bias and supports evidence-informed practice
Lack of economic evaluations	What are the economic impacts of different models of care and services for NVP and HG?	Supports resource allocation and health policy decisions
Inconsistent definitions and outcome measures in NVP and HG research	What NVP and HG definitions and outcome measures should be used in future research?	Supports comparability and synthesis of research findings

As expected, much of the literature was observational, including quality improvement projects focused on improving quality of care for affected women, and reducing hospital admissions and lengths of stay. Despite a strong focus on reducing inpatient admissions and length of stay in hospital MoC, there were only two cost utility analyses [38, 50] and another four studies that estimated cost savings [34, 55, 64, 69]. Future research should seek to incorporate a heath economic evaluation component to further assess the value of new or adapted NVP or HG MoC.

The predominance of observational studies is in keeping with the limited use of patient reported measures, with over one third of identified evaluations not including PROMs or PREMs. Where studies included PROMs or PREMs, PUQE was the most frequently reported, however there was wide variation in the tools used. International consensus on a core outcome set for HG research has been reached, which includes HG symptoms, laboratory findings, symptom management, service utilisation, quality of life and wellbeing including patient satisfaction with treatment, maternal harm, pregnancy complications, termination of pregnancy, birth outcomes and offspring outcomes [103]. Whilst this outcome set describes what to measure, the authors have acknowledged that more work needs to be done to establish recommendations for researchers on how to measure these outcomes [103].

To avoid the need for hospital admission, clinical practice guidelines in the UK, USA, Australia and New Zealand recommend outpatient services and in-home services for women who require parenteral fluid resuscitation and antiemetics [1, 9, 104]. This aligns with literature findings in this review, where outpatient management was associated with patient outcomes similar to those treated as inpatients [43, 44, 46], outpatient management resulted in reduced staff and economic burden on hospital inpatient wards [46, 47, 49, 50, 66], and women were satisfied with, and in some cases preferred, outpatient care [48, 49, 65]. Due to a lack of published evaluations, this review did not uncover any evidence to suggest that providing in-home care for women with NVP or HG is feasible. Evaluations of in-home care feasibility have been raised in the UK clinical guidelines as a recommendation for future research [9].

Much of the literature on telephone counselling support services was observational, and therefore did not assess any impact on quality of life or symptoms of women with NVP and HG. There were a small number of experimental trials that all demonstrated positive outcomes, however further evaluation of existing services is required to confirm that these impacts translate to real world settings. One area requiring further research lies in the investigation of differences in patient outcomes and satisfaction with care provided for women with HG and women with mild to moderate NVP. Early evidence from one study on weekly telephone counselling included in this review had identified reduced duration and severity of symptoms in women with mild or moderate NVP, but not in severe NVP [77]. Furthermore, all identified telephone counselling experimental trials excluded women with HG [75–78].

Other key gaps in the literature included initiatives to improve the management of NVP and HG in primary care settings, and social support services. The need for greater focus in this area is supported by current guidelines, which acknowledge the critical role that GPs and community pharmacists can play. This is particularly relevant in the management of NVP, which is common in early pregnancy, and may occur before specific pregnancy and birthing healthcare professionals have been engaged [9, 104]. Limited research and quality improvement in primary care may be a product of barriers to clinical governance in this sector, including the separation of primary care from other sectors, resource constraints, hierarchical structures, and a lack of external control over primary care organisations [105].

This systematic scoping literature review has comprehensively mapped the evidence for health care delivery arrangements for NVP and HG. Whilst study designs were categorised to consider the quality of evidence, full quality appraisal conducted as part of topic specific systematic reviews would provide further depth of understanding of the research available on this topic. Considering an identified need for more training, future reviews could also incorporate search terms to identify health care professional training programs [15, 65, 106]. As definitions of HG in clinical trials vary [107] and methods used to classify study participants were not always reported, the categorisation of study focus on NVP and/or HG in this review may not have applied consistent criteria. Finally, there may have been some MoC or services not identified in this review, either because they support all pregnant women and are not specifically targeted at women with NVP/HG, or because there was not sufficient evaluation data or information about an evaluation being conducted to qualify for inclusion.

## Conclusion

The aim of this scoping review was to map existing literature that evaluated health and social care delivery arrangements for women with NVP and HG, with a need for more original research on approaches to care provision identified. Where improvements on existing or new outpatient hospital management pathways had been implemented, positive patient outcomes and experiences and reduced length of hospital stays were reported. However much of this research was observational and limited to the UK context, indicating gaps in experimental or quasi-experimental research conducted across a range of health settings, with the potential to focus on PROMs and/or PREMs. Conference abstracts on quality improvement projects were prominent in the review findings, suggesting that there may be opportunities for further collaboration between researchers, hospital departments and health service providers to publish their work. Other research gaps identified include evidence for the effectiveness of primary care, social support and in-home care services, as well as economic evaluations. Lastly, guidelines to support a consistent approach to defining NVP and HG, and for measuring patient outcomes and experiences in research are needed.

## Supplementary Information

Additional File 1: PRISMA-ScR checklist.

Additional File 2: Search and screening strategy.

Additional File 3: Included records and extracted data.

## Data Availability

No datasets were generated or analysed during the current study.

## Abbreviations

GP: General Practitioner
HG: Hyperemesis Gravidarum
HiTH: Hospital in the Home
MoaH: Medical obstetrics at home
MoC: Model of Care
NVP: Nausea and Vomiting in Pregnancy
PROMs: Patient reported outcome measures
PREMs: Patient reported experience measures
PUQE: Pregnancy-Unique Quantification of Emesis Scale
RCT: Randomised controlled trial
