# Supplementary material for: A scoping review of models of care and services for nausea and vomiting in pregnancy and hyperemesis gravidarum
Source: BMC Pregnancy Childbirth. 2025 Oct 3;25:1012. doi: 10.1186/s12884-025-08093-y (PMC12495835; doi:10.1186/s12884-025-08093-y)
Supplement: Supplementary file 2 — Additional File 2: Search and screening strategy. [file 12884_2025_8093_MOESM2_ESM.docx]

**Additional File 2: NVP and HG Models of Care Scoping Review Search and Screening Strategy**

**Initial search run 2.8.23**

| Database | Search scope | Total | Total from Jan 2012 – 2.8.23 |
| --- | --- | --- | --- |
| Medline | (exp Morning Sickness/ or "morning sickness".mp. or "hyperemesis gravidarum".mp. or ((nausea or vomit* or emesis) adj3 (pregn* or matern* or gravid* or antenatal* or prenatal* or gestat*)).mp.) and (model or care or team or pathway* or service* or continuity or management or program* or practice or intervention or clinic).mp. | 1136 | 643 |
| Embase | (exp Morning Sickness/ or "morning sickness".mp. or "hyperemesis gravidarum".mp. or ((nausea or vomit* or emesis) adj3 (pregn* or matern* or gravid* or antenatal* or prenatal* or gestat*)).mp.) and (model or care or team or pathway* or service* or continuity or management or program* or practice or intervention or clinic).mp. | 2433 | 1567 |
| CINAHL | 1. (MH "hyperemesis gravidarum") or "hyperemesis gravidarum" or "morning sickness"  2. (nausea or vomit* or emesis) N3 (pregn* or matern* or gravid* or antenatal* or prenatal* or gestat*)  3. 1 OR 2  4. model or care or team or pathway* or service* or continuity or management or program* or practice or intervention or clinic  5. 3 AND 4 | 747 | 439 |
| PsycInfo | ("morning sickness".mp. or "hyperemesis gravidarum".mp. or ((nausea or vomit* or emesis) adj3 (pregn* or matern* or gravid* or antenatal* or prenatal* or gestat*)).mp.) and (model or care or team or pathway* or service* or continuity or management or program* or practice or intervention or clinic).mp. | 126 | 64 |
| Maternity and Infant Care | ("morning sickness".mp. or "hyperemesis gravidarum".mp. or ((nausea or vomit* or emesis) adj3 (pregn* or matern* or gravid* or antenatal* or prenatal* or gestat*)).mp.) and (model or care or team or pathway* or service* or continuity or management or program* or practice or intervention or clinic).mp. | 456 | 196 |
| Cochrane Library | 1. Exp Morning Sickness (MeSH) 2. ((nausea or vomit* or emesis) NEAR/3 (pregn* or matern* or gravid* or antenatal* or prenatal* or gestat*)) 3. “hyperemesis gravidarum” or “morning sickness” 4. #1 or #2 or #3 5. model or care or team or pathway* or service* or continuity or management or program* or practice or intervention or clinic 6. #4 and #5 | 433 | 334 |
| Scopus (title, abstract and keyword) | "hyperemesis gravidarum" OR "morning sickness" OR nausea OR vomit* OR emesis W/3 pregn* OR matern* OR gravid* OR antenatal* OR prenatal* OR gestat* AND model OR care OR team OR pathway* OR service* OR continuity OR management OR program* OR practice OR intervention OR clinic | 2281 | 1340 |
|  | TOTAL (before duplicates removed) |  | 4583 |
|  | TOTAL (duplicates removed in EndNote and Covidence) |  | 2303 |

**Updated search repeated 19.3.24**

| Database | Search scope | Total | Total from Jan 2023 – 19.3.24 |
| --- | --- | --- | --- |
| Medline | (exp Morning Sickness/ or "morning sickness".mp. or "hyperemesis gravidarum".mp. or ((nausea or vomit* or emesis) adj3 (pregn* or matern* or gravid* or antenatal* or prenatal* or gestat*)).mp.) and (model or care or team or pathway* or service* or continuity or management or program* or practice or intervention or clinic).mp. | 1183 | 91 |
| Embase | (exp Morning Sickness/ or "morning sickness".mp. or "hyperemesis gravidarum".mp. or ((nausea or vomit* or emesis) adj3 (pregn* or matern* or gravid* or antenatal* or prenatal* or gestat*)).mp.) and (model or care or team or pathway* or service* or continuity or management or program* or practice or intervention or clinic).mp. | 2564 | 220 |
| CINAHL | 1. (MH "hyperemesis gravidarum") or "hyperemesis gravidarum" or "morning sickness"  2. (nausea or vomit* or emesis) N3 (pregn* or matern* or gravid* or antenatal* or prenatal* or gestat*)  3. 1 OR 2  4. model or care or team or pathway* or service* or continuity or management or program* or practice or intervention or clinic  5. 3 AND 4 | 660 | 15 (Aug 2023 to 19.3.24) |
| PsycInfo | ("morning sickness".mp. or "hyperemesis gravidarum".mp. or ((nausea or vomit* or emesis) adj3 (pregn* or matern* or gravid* or antenatal* or prenatal* or gestat*)).mp.) and (model or care or team or pathway* or service* or continuity or management or program* or practice or intervention or clinic).mp. | 133 | 4 |
| Maternity and Infant Care | ("morning sickness".mp. or "hyperemesis gravidarum".mp. or ((nausea or vomit* or emesis) adj3 (pregn* or matern* or gravid* or antenatal* or prenatal* or gestat*)).mp.) and (model or care or team or pathway* or service* or continuity or management or program* or practice or intervention or clinic).mp. | 468 | 21 |
| Cochrane Library | 1. Exp Morning Sickness (MeSH) 2. ((nausea or vomit* or emesis) NEAR/3 (pregn* or matern* or gravid* or antenatal* or prenatal* or gestat*)) 3. “hyperemesis gravidarum” or “morning sickness” 4. #1 or #2 or #3 5. model or care or team or pathway* or service* or continuity or management or program* or practice or intervention or clinic 6. #4 and #5 | 647 | 29 (Aug 2023 – 19.3.24) |
| Scopus (title, abstract and keyword) | "hyperemesis gravidarum" OR "morning sickness" OR nausea OR vomit* OR emesis W/3 pregn* OR matern* OR gravid* OR antenatal* OR prenatal* OR gestat* AND model OR care OR team OR pathway* OR service* OR continuity OR management OR program* OR practice OR intervention OR clinic | 2153 | 167 |
|  | TOTAL (before duplicates removed) |  | 547 |
|  | TOTAL (additional duplicates removed in Covidence and EndNote) |  | 307 |

**Grey Literature Search Strategy**

| Country | Search scope | Date | Total |
| --- | --- | --- | --- |
| Australia | pregnancy OR pregnant AND "morning sickness" OR hyperemesis AND program OR service OR “model of care” site:gov.au | 28/2/24 | 50 |
|  | pregnancy OR pregnant AND "morning sickness" OR hyperemesis AND program OR service OR “model of care” site:org.au | 28/2/24 | 50 |
| New Zealand | pregnancy OR pregnant AND "morning sickness" OR hyperemesis AND program OR service OR “model of care” site: .nz | 21/2/24 | 55 |
| United Kingdom | pregnancy OR pregnant AND "morning sickness" OR hyperemesis AND program OR service OR “model of care” site: gov.uk | 21/2/24 | 17 |
|  | pregnancy OR pregnant AND "morning sickness" OR hyperemesis AND program OR service OR “model of care” site: org.uk | 29/2/24 | 50 |
| United States | pregnancy OR pregnant AND "morning sickness" OR hyperemesis AND program OR service OR “model of care” site: .gov | 22/2/24 | 50 |
|  | pregnancy OR pregnant AND "morning sickness" OR hyperemesis AND program OR service OR “model of care” site: .org | 22/2/24 | 50 |
| Canada | pregnancy OR pregnant AND "morning sickness" OR hyperemesis AND program OR service OR “model of care” site: .ca | 22/2/24 | 50 |
|  | Total |  | 372 |

**Literature screening questions:**

1. Is this paper focused on health or social care for women with NVP or HG? (proceed if ‘yes’)
2. Does the paper describe a model of care, program or service for women with NVP or HG? (proceed if ‘yes’). Noting:
   - A “Model of Care” broadly defines the way health services are delivered. It outlines best practice care and services for a person, population group or patient cohort as they progress through the stages of a condition, injury or event.
   - Excluding research specific to individual therapies (e.g. medications, other therapies such as IV fluids on patient outcomes), including care pathways and services including models of care where services can be tailored (but not necessarily) to patients needs. Interested in health-system level initiatives rather than individual therapies – i.e. exclusion of clinical studies related to informing individual health care provider clinical decision making (e.g., testing effectiveness of medications).

Additional questions added through discussion and consensus amongst the research team:

1. Is there an evaluation of the model of care, program or service? (proceed if ‘yes’) Noting a planned evaluation can be included (e.g., trial registration, trial protocol paper)
2. Is the model of care, program or service focused on a health or social care delivery arrangement (i.e., how care is designed to meet consumer’s needs, by whom care is provided, where care is provided and with what supports care is provided)? (include paper if ‘yes’)
