# Supplementary material for: A scoping review of models of care and services for nausea and vomiting in pregnancy and hyperemesis gravidarum
Source: BMC Pregnancy Childbirth. 2025 Oct 3;25:1012. doi: 10.1186/s12884-025-08093-y (PMC12495835; doi:10.1186/s12884-025-08093-y)
Supplement: Supplementary file 3 — Additional File 3: Included records and extracted data. [file 12884_2025_8093_MOESM3_ESM.docx]

**Additional File 3: Included records and extracted data**

**Hospital models of care** (n = 31 MoC from 34 records)

Outpatient management (n = 15 MoC from 15 records)

| **Description (ref)** | **Country** | **Study design and population** | **Aim** | **Outcome measures and data sources (incl. PROMs/PREMs)** | **Key findings** | **Type of publication and other notes/ limitations** |
| --- | --- | --- | --- | --- | --- | --- |
| Introduction of four new guidance documents including flowchart for community practitioners, new maternity assessment unit (MAU) guideline, MAU assessment sheet, and GP communication sheet.  **Buchanan et al 2016 (1).** | UK | Retrospective audit, QI and re-audit  Observational (QI)  NVP | To promote outpatient management of acute NVP through audit and quality improvement | Admission and readmission rates, completion rates of new assessment sheets and compliance with g uideline protocols.  No PROMs or PREMs | Admission rates decreased with the use of new guidance documents however ongoing staff training to improve clinical protocol adherence was required. | Abstract |
| New day case hyperemesis unit pathway with a day case bay with recliner chairs. Rapid rehydration protocol over 6-8 hours alongside antiemetics, discharge same-day, open access self-referral, education of staff.  **Channing et al 2021 (2).** | UK | QI and audit  Observational (QI)  HG | To manage at least 40% of eligible pregnant women referred with severe NVP through day case pathway | Number and percentage of women able to be managed in the day case pathway.  No PROMs or PREMs | Achieved and maintained target of managing 40% of eligible women. Limiting factor for implementation was lack of availability of day case bay (other uses). | Abstract |
| Hydration unit (HU) within the hospital (designated area on the ward where patients receive treatment without occupying an acute bed). Gynaecology Senior House Officer (SHO) education and alteration of HU opening times from 9am-5pm to 11 am-7 pm was initiated between audits.  **Cobb et al 2014 (3).** | UK | QI with clinical audit and re-audit  Observational (QI)  HG | To improve compliance with the Hyperemesis Gravidarum Trust guideline | Prospective collection of patient personal and demographic data.  No PROMs or PREMs | Admission to the HU increased from 16.6% to 37.5%. By avoiding admission to the ward, £5750 was saved on inpatient bed days over the 5-month period. | Abstract |
| Implementation of a novel day case pathway for patients with severe nausea and vomiting in pregnancy at the Gynaecology Emergency Clinic (GEC). This facility is open from 9 am to 5 pm on weekdays where dedicated nursing staff and clinicians provide patients with antiemetics, fluids, and appropriate care. A 'hyperemesis pack' containing paperwork for prescribing and patient information leaflets was developed.  **Fernandopulle and Doraiswamy 2021 (4).** | UK | QI with retrospective audit  Observational (QI)  HG | To assess the appropriateness of referrals from General Practitioners and Accident and Emergncy to the GEC and onto the ward in line with national guidelines. | Patient record review to analyse care that was received.  PROMs – PUQE score was not recorded in 94% of admissions (PUQE version not specified) | Patients attending outside GEC opening hours were more likely to be admitted. Directing referrals to GEC reduced pressures on Accident and Emergency. Referring patients during GEC opening hours reduced the likelihood of overnight admissions. | Abstract |
| Implementation of the Rapid Hydration Clinic for hyperemesis patients within the Emergency Gynaecology Unit. Outpatient management of hyperemesis gravidarum using a Fast Hydration Protocol.  **Ijaz et al 2016 (5).** | UK | Observational  HG | To assess the impact of the Rapid Hydration Clinic on inpatient admission rate. | Demographics, gestation, number of attendances, electrolyte status, ultrasound results, requirement of antiemetics and fluids, and indication for inpatient treatment were collected from the Emergency Gynaecology Unit register for patients managed as outpatients or admitted for hyperemesis.  No PROMs or PREMs | The admission rate decreased from 73% to 10%. | Abstract |
| Hyperemesis Day Centre (HDC) - Outpatient nurse run protocol for HG where patients are fast hydrated over a period of a few hours and then discharged.  **Khan et al 2013 (6).** | UK | Observational  HG | To assess if the HDC protocol was being followed, and if inpatient admissions were reduced. | Patient case notes including HDC pro forma in notes and patient outcomes.  PREMs (no details provided). | The centre successfully reduced inpatient admissions, improved patient experience and is safe and efficient. | Abstract |
| Multidisciplinary HG day case service (IRIS hydration clinic) established in May 2020. It includes care, treatment and support from midwives, dietitians, obstetricians and mental health professionals. It runs every Tuesday afternoon, with six women attending the clinic at a time.  **Doherty et al 2023 (7).** | Ireland | Qualitative (interviews)  Observational  HG | To explore women’s experiences of HG and of attending the clinic. | Key themes identified from interview data.  No PROMs or PREMs | There was an appreciation for a dedicated clinic, rather than receiving ad hoc treatment. Relationships were important where women described the benefits such as continuity of care and the positive impact of peer support. Areas for improvement included more sensitivity around women’s issues such as weight gain/loss. | Full text |
| IRIS (Intravenous fluids, Rest, Insight and Support) hydration clinic as above.  **O’Brien et al 2023 (8).** | Ireland | Sequential, mixed-methods study  Observational and Quasi-experimental (pre-post test)  HG | To explore the effectiveness and feasibility of the clinic via retrospective chart review, pre-test-post-test to assess symptoms of HG, well-being, food tolerances, QoL and nutritional intake of 50 women 8 weeks post intervention, and semi-structured interviews to explore women’s experiences of HG and attending the IRIS clinic. | Chart review to describe assessments, treatments, pregnancy and birth outcomes.  Qualitative – semi structured interviews with women on their experiences of attending the clinic.  Primary outcome was change in PUQE score. Secondary outcomes included maternal subjective well-being, quality of life, gestational weight gain, dietary intakes and infant birthweight.  PROMs   - PUQE score obtained in retrospective chart review and also collected as part of the pre-post study to assess symptom severity (PUQE-24) - Pregnancy Symptoms Inventory (PSI) - 12-item short form health survey (SF-12) - WHO-5 Well-being index | N/A | Protocol |
| New ambulatory care pathway implemented for NVP. Quality improvement included staff education and training, updated guidelines, developing a patient assessment and management proforma and establishing an ambulatory care service.  **Pilling et al 2018 (9).** | UK | QI and audit cycle  Observational (QI)  NVP | To present a completed audit cycle, which outlines changes in practice in a single centre after implementing a standardised care pathway and development of ambulatory care. | Admission data for comparison against 29 specific quality indicators in the Royal College of Obstetricians and Gynaecologists (RCOG) guidelines.  No PROMs or PREMs | Re-audit demonstrated a reduction in hospital admissions (38% versus 100%) and mean length of stay (0.6 days versus 1.6 days), alongside improved compliance with 11/29 clinical quality indicators. | Abstract |
| Ambulatory care bundle for the treatment of women suffering with symptoms relating to hyperemesis gravidarum (HG). The bundle includes IV fluid hydration, patient information, and discharge with cyclizine, thiamine and folic acid.  **Tompsett et al 2013 (10).** | UK | Observational  HG | To reduce the number of admissions for HG. | Admissions data.  PROMS – PUQE was used clinically as part of the care bundle (PUQE version not specified) | The number of monthly admissionsand overall length of stay reduced | Abstract |
| The Ambulatory Hyperemesis Unit (AHU) provides outpatient management for pregnant women with HG. It is available between 9am-1pm Monday-Friday and is run by nursing staff.  **Ucyigit 2020 (11).** | UK | Observational (12 years)  Estimated cost saving  HG | To investigate the impact of the AHU on inpatient admission rate and length of stay for hyperemesis gravidarum, and to explore the financial impact. | Admissions data.  No PROMs or PREMs | There was a significant reduction in the length of stay per patient admission and the total number of inpatient nights per year. This equated to an estimated cost-saving of almost £100,000 per year. The total number of inpatient admissions did not change. | Full text |
| Ambulatory Hyperemesis Gravidarum Initiative. Improvement strategies were devised in response to admission, treatment and clinical outcome audit data comparing practice to clinical guidelines in addition to patient experience survey feedback.  **Wang and Ma 2018 (12).** | UK | QI and clinical audit with patient experience survey  Observational (QI)  HG | To implement and evaluate the impact of an outpatient ambulatory approach to managing HG on admission, re-admission rates and patient satisfaction. | Number of admissions, readmissions rate and patients’ experiences survey.  PREMs – patient experience survey (no further details provided) | An 18% reduction in the rate of hospital admission was achieved alongside a low readmission rate (<0.5%). All patients were satisfied with the service and would recommend to others. | Abstract |
| Day admission service (DAS) for HG established in 2007 underpinned by robust evidence-based practice guidelines and patient information leaflets.  **Ajufo 2013 (13).** | UK | Observational (4 years)  HG | Assessing the impact of HG DAS on subsequent local occurrence and admissions. | Impact of DAS was derived indirectly from monthly and yearly attendance data for HG  -Length of hospital stay  -Information received by patients  -Staff support given  Retrospective data collection through local pregnancy database.  No PROMs or PREMs | Of 232 patients identified, 14.7% were treated through DAS, 43.5% as outpatients and 41.8% via hospital admission. Patient numbers in all treatment groups declined over the four-year period, with the largest reduction seen in inpatient numbers. Ward patients recorded longer hospital stay in days compared to hours for DAS. | Abstract |
| Outpatient hyperemesis service.  **Clark 2016 (14).** | UK | Audit and QI  Observational (QI)  HG | To report successes, failures, staffing profiles and results of auditing individual care pathways and learnings for others seeking to set up a similar service. | Not reported  No PROMs or PREMs | The hyperemesis outpatient service contributed to a reduction in inpatient stay. | Abstract |
| Day Case Service for treatment of HG (secondary care without overnight stay). Patients receive blood tests, IV hydration, thiamine and 4 hourly observations.  **Coleman et al 2014 (15).** | UK | Mixed methods  Observational  HG | To identify whether the day case service reduces hospital admissions, assess protocol compliance and evaluation patient satisfaction. | Patient audit (hospital admissions and protocol compliance check), patient satisfaction survey.  PREMS – Patient satisfaction surveys (no further details provided) | Out of the 13 patients audited, 7 were admitted, suggesting that the ambulatory care service reduced hospital admissions by 46%. Patient satisfaction surveys indicated that 100% of patients felt better after their day care treatment, and 94% would choose the same treatment again. Further staff training is required to improve rates of baseline observations and thiamine treatment. | Abstract |

Inpatient management (n = 4 MoC from 5 records)

| **Description (ref)** | **Country** | **Study design and population** | **Aim** | **Outcome measures and data sources (incl. PROMs/PREMs)** | **Key findings** | **Type of publication and other notes/ limitations** |
| --- | --- | --- | --- | --- | --- | --- |
| Patient assessment using Hyperemesis Impact of Symptoms Questionnaire to plan an individualised care plan and ongoing support and advice from a research nurse. This extends beyond rehydration and antiemetic therapy (usual care), with nurses and midwives taking a more personalised approach that validates women’s experiences and provides targeted reassurance and practice advice to support each woman to develop strategies to manage symptoms following discharge from hospital. Tailored advice included: dietary advice, practical advice regarding symptom management and counselling regarding psychological impact of symptoms. Opportunity to follow up via phone for advice following discharge was offered.  **Fletcher et al 2013; Fletcher et al 2015 (16, 17).** | UK | RCT  Cost-effectiveness  HG | Psychosocial effect and cost-effectiveness of holistic assessment and tailored plan | Demographic data, hospital admission data  PROMs   - Hyperemesis Impact of Symptoms Questionnaire as part of clinical care - Short form health survey (SF-36) - EQ5D - PUQE (PUQE-12)   PREMs – Client Satisfaction Questionnaire (CSQ)  Primary outcome: Social functioning at 2 weeks follow up (one subscale of the SF-36)  Secondary outcome: Health status, nausea and vomiting, satisfaction and economic outcomes.  QALY weighted by EQ5D | Women’s average social functioning, Hyperemesis Impact of Symptoms scores and average number of admissions were not significantly different between either group. Average number of days in hospital was significantly lower for the intervention (4.97) compared to the control group (6.14). Using a tailored plan of care to address women’s individual needs was not associated with any significant improvement in QoL measures, nor was it cost saving. | Full text  Abstract |
| A proforma that was developed by a multidisciplinary team. ​ The proforma served as a tool for junior doctors to prompt appropriate investigations, medications, and fluid resuscitation for patients with HG. ​It included a clerking sheet for the patient, space to document blood results, a fluid resuscitation regime, a checklist for antiemetics, and prompts for prescribing folic acid, thiamine, and venous thromboembolism prophylaxis.  **Lloyd et al 2014 (18).** | UK | QI and audit  Observational (QI)  HG | Improve the management of patients with hyperemesis gravidarum by addressing issues related to fluid resuscitation, prescribing practices, and length of admission. The study aimed to implement a proforma and evaluate its impact on patient care. ​ | The study evaluated the impact of the proforma on patient care by comparing baseline measurements to post-measurements. The outcomes of fluid resuscitation, prescribing practices, and length of admission were assessed. ​  No PROMs or PREMs | Outcomes included improvements in fluid resuscitation, prescribing practices, and the use of specific medications for patients with HG. ​There was an increase in the volume of fluid given over the first 24 hours, 100% potassium replacement, and improved prescribing of thiamine, folic acid, and enoxaparin. ​The proforma was well-received by junior doctors. | Full text |
| Implementation of a new initial management protocol for NVP/HG aimed at improving the quality of care and reducing length of hospital stay. The protocol included elements such as rapid rehydration, anti-emetic administration, and thiamine prescription.  **Nam et al 2018 (19).** | UK | QI and audit  Observational (QI)  NVP/HG | To assess the effectiveness of the new management protocol for NVP/HG in reducing the length of hospital stay and improving the quality of care provided to patients. | The main outcome measured was the average length of hospital stay for patients with NVP/HG.  Admissions with NVP/HG were recorded in a 'Hyperemesis audit' book, and a proforma of the new initial management protocol was provided.  No PROMs or PREMs | The results indicated that the new protocol contributed to reducing the average length of hospital stay for patients with NVP/HG. However, only a portion of admissions had the proforma fully completed, suggesting areas for improvement in protocol adherence. | Abstract |
| The study utilised the Pregnancy-Unique Quantification of Emesis (PUQE) questionnaire to assess the severity of nausea and vomiting of pregnancy (NVP) in hospitalised women with HG.  **Laitinen et al 2022 (20).** | Finland | Prospective cohort study  Observational  HG | To evaluate the usability of the Finnish-translated PUQE in hospitalised women with HG. | The study assessed categorised and continuous PUQE scores, physical and mental quality of life (QoL) (measured using visual analogue scales (VAS)), and urine ketones at admission and discharge, analysing the first admission and readmissions separately.  PROMs – PUQE (PUQE-12)  Two VAS to assess physical and mental QoL | The PUQE scores reflected alleviation of NVP severity in women hospitalised due to HG. Better physical QoL was associated with lower PUQE score category, indicating less severe NVP. PUQE is recommended as a complementary instrument in the inpatient setting. | Full text |

Comparison of outpatient and inpatient management (n = 8 MoC from 10 records)

| **Description (ref)** | **Country** | **Study design and population** | **Aim** | **Outcome measures and data sources (incl. PROMs/PREMs)** | **Key findings** | **Type of publication and other notes/ limitations** |
| --- | --- | --- | --- | --- | --- | --- |
| The study compared ambulatory (outpatient) treatment of severe NVP with inpatient care. ​Ambulatory care involved daily attendance for treatment in an ambulatory care unit or gynaecology ward area, while inpatient care involved continuous supportive care in a gynecology ward.  **Mitchell-Jones et al 2017; Mitchell-Jones et al 2017; Isrctn 2014 (21-23).** | UK | RCT with a patient preference arm  HG | To determine whether ambulatory treatment of severe NVP is as effective as inpatient care. ​ | The primary outcome measure was the reduction in Pregnancy Unique Quantification of Emesis (PUQE) score 48 hours after starting treatment. ​ Secondary outcome measures included the duration of treatment, improvement in symptom scores and ketonuria at 48 hours, reattendances within 7 days of discharge, and comparison of symptoms at 7 days post-discharge.  PROMS – PUQE score (PUQE-12)  Wellbeing rating  (0-10)  Eating and drinking scoring system (1-5). | Ambulatory management was found to be an effective alternative to inpatient management of severe NVP. ​There was no significant difference in the reduction of PUQE score at 48 hours between the ambulatory and inpatient treatment groups. ​Both treatment options were effective in improving symptom scores and ketonuria. ​The duration of treatment and reattendance rates within 7 days were also similar between the two groups. ​ | Abstract  Full text  Trial registration |
| The model of care was midwifery-led day-case management of severe NVP, which involved rapid rehydration, symptom relief, and ongoing midwifery telephone support.  **McParlin et al 2016 (24).** | UK | Pilot RCT  HG | To evaluate the feasibility of the intervention and compare outcomes between the intervention (day case management) and control (inpatient care) groups. | The outcomes of interest were improvements in objectively assessed NVP, quality of life scores, satisfaction with care, obstetric and neonatal outcomes, readmission rates, completion rates, and length of hospital stay. ​  PROMs -  Pregnancy Unique Quantification of Emesis and Nausea (PUQE) score (PUQE-12),  Short Form 36 version 2 (SF36.v2) Physical and Mental component scores  PREMs - Satisfaction score. ​Harvey S, Rach D, Stainton MC, Jarrell J, Brant R. Evaluation of satisfaction with midwifery care. Midwifery 2002;18(4):260–7. | Improvements in NVP, QoL and satisfaction with care were similar between groups. ​Overall time in the hospital was substantially reduced in the intervention group. ​A larger trial was deemed feasible, as 70% of eligible women agreed to be randomised. ​A low completion rate of follow-up questionnaires was reported. | Full text  RCT |
| The study compared day care (outpatient) treatment with inpatient management for pregnant women with nausea and vomiting of pregnancy.  **McCarthy et al 2014 (25).** | Ireland | RCT  NVP | To evaluate the differences between day care and inpatient management of pregnant women with nausea and vomiting of pregnancy. ​ | The primary outcome was the total number of inpatient nights related to nausea and vomiting of pregnancy. ​ Secondary outcomes included quality of life, total volume of intravenous fluids administered, total amount of antiemetics administered, total multivitamin complexes administered, and patient satisfaction. ​  PROMs:  EQ-5D  PREMs - Client Satisfaction Questionnaire | Treatment of NVP in day care reduces hospital inpatient stay compared with inpatient treatment and day care was satisfactory to patients. | Full text |
| The intervention involved day care management of nausea and vomiting of pregnancy (NVP), which included initial treatment with intravenous fluid administration and stepwise administration of antiemetics. By contrast, inpatient management included intravenous fluid administration and antiemetic administration, with patients staying in the hospital until they could tolerate oral fluids.  **Murphy et al 2016 (26).** | Ireland | Cost-utility analysis  NVP | To evaluate the cost-effectiveness of day care management compared to inpatient management of NVP using a decision analytical model. | The main outcomes measured were total costs and quality-adjusted life years (QALYs) associated with day care and inpatient management of NVP.  PROMs – QALY’s derived from EQ-5D and SF-36 scores in the literature. | Day care management of NVP was found to be less costly and more effective compared to inpatient management. Specifically, day care management was associated with lower costs and higher QALYs compared to inpatient management. | Full text |
| The study compared treatment for HG in a day case setting (HGDU) to hospital admission. HGDU refers to any outpatient style treatment for HG within a hospital or clinic where a woman can receive rapid IV rehydration and medication during the day and return home overnight.  **Dean and Marsden 2017 (27).** | UK | Cross-sectional survey (online survey)  Observational  HG | To establish if treatment for HG in a day case setting is associated with improved satisfaction compared to a hospital admission. | The main outcome of interest was the level of satisfaction with treatment for HG in a day case setting compared to hospital admission.  PREMs – Satisfaction questionnaire developed in collaboration with key stakeholders and the literature. | Satisfaction was significantly greater among the HGDU group. There were no differences in the medical treatment received, however satisfaction was greater in the HDGUs for information provision, levels of staff knowledge and feeling better on discharge. HDGUs were associated with a significant reduction in the number of days spent in hospital and therefore represented cost savings. | Full text |
| HG Day Units (HGDU) situated within early pregnancy units in the hospital setting (outpatient service) where women can receive rapid IV rehydration and medication.  **Dean and Marsden 2017 (28).** | UK | Qualitative component of a larger scale cross-sectional survey  Observational  HG | To explain why treatment for HG in a day case setting is associated with improved satisfaction compared to a hospital admission | Reasons for patient satisfaction and dissatisfaction of HGDU and hospital care for HG.  PREMs – Satisfaction questionnaire developed in collaboration with key stakeholders and the literature. | The need for staff education was highlighted by women from both MoC and better information for patients and understanding and validation from staff. A perceived benefit of a day unit was that treatment would be received early and more efficiently. For most women, having children at home was a reason for preferring a HGDU, with many preferring a bed rather than a chair. Staff understanding (regardless of knowledge level) was critical to women’s wellbeing. | Full text |
| Day case management (DCM) involved referral to an on-call physician, initial investigations, and treatment initiation in an ambulatory clinic setting, while inpatient management (IPM) involved admission to the ward for treatment.  **Ryan et al 2019 (29).** | UK | Observational (1 year)  HG | To investigate the total length of stay, comparing those who were managed as inpatients as opposed to those managed in a day case setting. | Patient satisfaction with DCM, re-admission rates, biochemical parameters.  PREMs – Patient satisfaction questionnaire  González N, Quintana JM, Bilbao A, et al. Development and validation of an in-patient satisfaction questionnaire. Int J Qual Health Care 2005; 17(6): 465-72. | Day case managed patients for HG had a significantly shorter length of stay and patient reported satisfaction was high. | Full text  Noted that use of the PUQE would have enhanced the study methodology. |
| Implementation of the Hyperemesis Ambulatory Rehydration Project (HARP) to minimise hospital stay for hyperemesis gravidarum patients. Outpatient management of dehydration using intravenous (IV) rehydration alongside anti-emetic medication. Patients discharged to HARP once urine demonstrates 2+ ketones or less and they tolerate oral fluids. Discharged patients receive IV fluids as outpatients until satisfactorily rehydrated. A HARP protocol was developed and outpatient management area identified.  **Hordern et al 2013 (30).** | UK | Audit and QI  Observational (QI)  HG | To determine the hyperemesis admission rate, hydration status of patients, length of stay, and re-admission rates in the unit and evaluate if outpatient management through HARP could reduce length of stay and re-admission rates. | Review of patient notes to determine inpatient hours, re-admission rates, medications prescribed on discharge and ketones on admission.  No PROMs or PREMs | Review of 100 hyperemesis admissions showed an average of 8.88 patients per month. HARP was offered to 40 patients, 35 accepted, and 27 notes available for review. Similar re-admission rates between inpatient and HARP groups were apparent, however the HARP group showed a reduced length of stay if re-admitted, suggesting some improvement in overall rehydration status. | Abstract |

In Home Care (n = 3 MoC from 3 records)

| **Description (ref)** | **Country** | **Study design and population** | **Aim** | **Outcome measures and data sources (incl. PROMs/PREMs)** | **Key findings** | **Type of publication and other notes/ limitations** |
| --- | --- | --- | --- | --- | --- | --- |
| Intravenous hydration treatment at home over 3 days via the HITH model.  **Canty et al 2022 (31).** | Australia | Mixed methods including pre and post-test survey and interviews.  Observational  Economic analysis  HG | To examine the effectiveness, feasibility, implementation and dissemination of intravenous hydration for moderate to severe HG via the HITH model. | The outcome of rates of representation (return visits) to the emergency department (ED) for women receiving HITH model of care for moderate to severe hyperemesis gravidarum (HG). The HITH model of care of economic analysis and implementation costs.  PROMs – PUQE score used clinically (PUQE version not specified)  QoL tools (no details provided  PREMs – Satisfaction survey (no details provided). | N/A | Protocol abstract |
| Medical Obstetrics @ Home (MOaH). Integrated care model for managing complex obstetric conditions at home with ongoing care via telehealth.  **Carlon and Kiroglu 2022 (32).** | Australia | QI  Observational (QI)  HG | Not specified | Not specified.  No PROMs or PREMs | N/A | Protocol abstract |
| Home treatment with intravenous fluids for selected patients was introduced as part of a relocation of HG care to the department of gynaecology. A patient manual for patients with home-administered fluids was developed in consultation with patients and staff.  **Ostenfeld et al 2023 (33).** | Denmark | QI  Observational (QI)  HG | Aims of this QI project included patient satisfaction with the relocation of HG care and number of follow ups conducted via phone. | Patient satisfaction and suggested improvements.  PREMs – Patient satisfaction questionnaire developed | Both (n=2) patients reported high satisfaction with the home treatments and expressed relief that they no long had to visit the hospital several times each week. Both patients however experienced complications due to the installed catheters after the project period. It was therefore agreed that a central venous catheter should be offered only as a last resort and that home treatment could also be administered for patients with midlines. | Full text |

Emergency department (n = 1 model from 1 record)

| **Description (ref)** | **Country** | **Study design and population** | **Aim** | **Outcome measures and data sources (incl. PROMs/PREMs)** | **Key findings** | **Type of publication and other notes/ limitations** |
| --- | --- | --- | --- | --- | --- | --- |
| Emergency Department (ED) short stay unit (CDU). A new clinical pathway (proforma) was developed collaboratively by emergency department (ED) and obstetrics and gynaecology (O&G) teams to optimise care of patients presenting with HG in ED and minimise unnecessary admissions to O&G.  **Skalley et al 2018 (34).** | UK | QI and audits  Observational (QI)  HG | To explore the impact on admission rates to O&G and patient care. | Patient management indicators (investigations, urinalysis, blood glucose, IV fluids, antiemetics, folic acid, thiamine, thromboprophylaxis), discharge outcome and length of stay.  No PROMs or PREMs | An improvement in prescriptions of antiemetics, vitamins and thromboprophylaxis was seen in line with the hospital guideline. Admissions to O&G were reduced by 68.7% (audit cycle 1) and 70.5% (audit cycle 2) compared with admission rates before the proforma. This was estimated to have saved £265,700 and 235 bed days over one year. | Full text |

**Digital Health** (n = 15 MoC from 19 records)

Telephone and web-based support (n = 12 MoC from 14 records)

| **Description (ref)** | **Country** | **Study design and population** | **Aim** | **Outcome measures and data sources (incl. PROMs/PREMs)** | **Key findings** | **Type of publication and other notes/ limitations** |
| --- | --- | --- | --- | --- | --- | --- |
| Motherisk NVP helpline. Counsellors and fellows take calls regarding exposure during pregnancy and lactation.  **Chua-Gocheco et al 2012 (35).** | Canada | Descriptive  Observational  NVP | To summarise calls to the helpline in 2011. | Total calls, calls for each line (including NVP service), type of caller, # questions per call, reason for call and geographical area.  No PROMs or PREMs | NVP had 2035 calls. Calls came from women and their partners (82%) and HCPs (18%). 99% were in Canada.  Other findings were reported for all Motherisk services rather than NVP line alone. | Abstract |
| Motherisk NVP helpline. Motherisk NVP Helpline, established in 1995 and provided toll-free in Canada and the USA.  **Madjunkova et al 2013; Madjunkova et al 2013 (36, 37).** | USA | Descriptive  Observational  NVP | To identify the primary concerns of American women contacting the Motherisk NVP Helpline, characterise the severity of their symptoms and describe the therapy offered. | Helpline data from Jan 2008- July 2012 to review intake forms for residence, age, gravidity, parity, PUQE score, and other available clinical characteristics including main reason for the call.  PROMs – PUQE (PUQE-24) | Of 195 forms reviewed, 86% called for management of NVP and 14% called solely about drug safety during pregnancy/ breastfeeding from all across the USA. Their leading concern was the use of doxylamine and vit B6 combination for NVP, followed by the use of ondansetron. Most women had a moderate PUQE score (77.12%, followed by severe (17.8%) and mild (5.08%). | Full text  Abstract |
| Motherisk NVP helpline.  **Madjunkova et al 2014 (38).** | USA | Descriptive  Observational  NVP | To describe antiemetic use among American women calling Motherisk NVP Helpline in Toronto from review of intake forms over last 10 years. | Clinical and demographic data with details on NVP treatment, symptoms and concomitant medical conditions.  PROMs – PUQE (version not specified) | Of 522 women 10.3% had mild, 77.7% had moderate and 12% had severe NVP. 15% had HG. 28% did not receive any antiemetic although 71.4% of them had moderate and 12.9% had severe NVP. Most frequent monotherapy was doxylamine (48%) followed by ondansetron (39%). | Abstract |
| Motherisk NVP helpline. Women with HG (intervention group) and women with varying degrees of NVP (control group) that contacted the Motherisk NVP helpline. Both groups received counselling on dietary changes, pharmacotherapy, and psychological support.  **Maltepe et al 2015 (39).** | Canada | Prospective cohort study  Observational  NVP/HG | To study the effects of counselling and predictors of pregnancy outcomes in women with HG compared to NVP | Comparison of age, gestation when support was sought, length and severity of symptoms, duration of gestation, birth weight and perinatal complications. Breastfeeding duration and infant developmental milestones.  No PROMs or PREMs | Outcomes were more favourable in women with HG than NVP. The number of counselling sessions per week was a significant predictor for maternal weight gain (p=0.001). Severity of NVP was a significant predictor for duration of gestation and child speech development (p=0.02 and 0.01). | Abstract |
| Garbha-Swasthya helpline. Run by tertiary care private hospital to address issues related to pregnancy and its complications. Established in 2010 and run voluntary. 10-4 pm in multiple languages.  **Godbole et al 2015 (40).** | India | Descriptive  Observational  NVP | To report on caller characteristics, number of calls and related information and the reasons to call the helpline. | Helpline data from Sep 2010 to Dec 2012 to understand caller characteristics, number of calls and related information, and reasons for call.  No PROMs or PREMs | 696 calls (275 were repeat callers). 73.3% pregnant women, 24.8% relatives, 1.9% medical professionals. All calls from HCPs related to drug safety in pregnancy. 27.4% of calls were about exposures (including drugs) and 14% about NVP. | Full text |
| University of North Carolina Health Care System Drug Information Center.  **Patil et al 2014 (41).** | USA | Descriptive  Observational  HG | To characterise pregnancy and lactation-related medication inquiries to identify classes of medication of most concern to providers and any trends in provider inquiries. | Pregnancy and lactation-related medication inquiries between Jan 2001-Dec 2010.  Provider type, date of inquiry, indication for medication inquiry, type of information requested.  No PROMs or PREMs. | 50% physicians, 21% pharmacists, 18% nurses. 34% were pregnancy related (vs. preconception and postpartum). Psychiatry and infectious diseases were the most common indicators for inquiry (obstetrics was 4^th^ and increased overtime, including HG). | Full text |
| SafeMotherMedicine medicines information service. A web-based medicines information service.  **Heitmann et al 2023 (42).** | Norway | Observational  NVP/HG | To characterise inquiries to describe the need for lactation risk information among women with NVP or HG. | Review of inquiries received between June 2011 and May 2022, which along with corresponding answers are stored in a full-text database. The database was searched for inquiries concerning antiemetics for NVP or HG during breastfeeding.  Medicine name, timing of medicine use with respect to breastfeeding, reason for asking SafeMotherMedicine, age of breastfed child.  No PROMs or PREMs | 97 inquiries addressing the use of antiemetics for NVP or HG during breastfeeding were identified, which addressed meclizine (51%), metoclopramide (33%), promethazine (16%), ondansetron (9%), and others (6%). SafeMotherMedicine was most often consulted before medicine use (63%), and in 49% of the inquiries, the women had already discussed the use with a physician. For one third of inquiries the contact with the service was caused by restrictive information about medicine safety in breastfeeding.  There is an information need about the use of antiemetics during lactation for women breastfeeding an older infant whilst suffering from NVP/HG. | Full text |
| e-Sanjeevani. A cloud-based web platform. OPD (patient to provider platform) and AB-HWC (provider-to-provider platform based on a hub and spoke model) exist with the latter being of interest in this study. The hub is the central point of medical expertise, and spokes connected to health workers and patients in remote areas. A health care worker to doctor (specialist) consultation regarding a patient. After interaction, provisional diagnosis and management plan is made, and an electronic prescription is generated and printed at the patient’s end. Operates 9am-4pm.  **Singh et al 2023 (43).** | India | Prospective observational  Observational  HG | To evaluate the clinical spectrum of patients presenting with obstetrical and gynaecological disorders, their management and the use of teleconsultation | Platform consultation records including reason for consultation, patient demographics, management plan details.  No PROMs or PREMs | 21.2% (n=682) of patients were antenatal. Some patients required referral to higher centres for admission, observation and fluid resuscitation, especially in hyperemesis gravidarum subgroup. | Full text |
| Nursing telephone support for women with NVP. Offering measures to improve diet and lifestyle as well as actions for reducing fatigue and enhancing psychological wellbeing via a 15-20 minute phone call twice per week for 4 weeks) vs control group (routine care).  **Abedian et al 2014; Irct2014012316326N 2014 (44, 45).** | Iran | RCT  NVP  Inclusion:  6-10 weeks gestation. First pregnancy. Mild-mod NVP (PUQE-24). Not using anti-nausea drugs | Effect of telephone support on the severity of NVP in the first trimester. | Demographic questionnaire, phone call log, PUQE-24.  PROMs – PUQE (modified PUQE) | Mean score of NVP was significantly different in experimental and control groups after intervention (p=0.028). Mean score of NVP in the experimental group were significantly different before and after the intervention (p<0.001), but not in the control group (P=0.272). Telephone support reduces severity of NVP. | Abstract  Trial registration |
| Nursing telephone support for social support twice a week for 4 weeks. Each phone call lasted approximately 15-20 minutes. Dietary and lifestyle changes, as well as ways to reduce fatigue and improve psycho-emotional status were discussed. Women could also call the researcher anytime between 8 am-8 pm.  **Abedian 2015 (46).** | Iran | RCT  NVP  Recruiting Primiparous women with NVP in the first half of pregnancy (criteria 6-10 weeks) with mild to moderate nausea as per the modified questionnaire of pregnancy-related nausea and vomiting (modified-PUQE). | Effect of telephone support on stress and perceived social support in primiparous women with NVP | Demographic questionnaire, multidimensional scale of perceived social support, visual analogue scale for stress, modified PUQE.  PROMs -  Modified PUQE  Stress related visual analogue scale (VAS)  Multidimensional scale of perceived social support (MSPSS) | No significant difference was found in the mean scores of stress in two groups at the beginning and end of the study. However, social support score at the beginning of the study was significantly different from that at the end of the study in the intervention group. It was recommended that telephone support be integrated into the routine care of women with NVP to improve their perceived social support and decrease stress. | Full text |
| Weekly telephone counselling. Follow-up counselling vs. standard outpatient clinic service (control). Weekly telephone counselling involved nursing interventions directed towards alleviating NVP using Roy’s adaptation model (RAM), provided until NVP terminates  **Isbir and Mete 2016 (47).** | Turkey | Quasi-experimental (non randomised)  NVP (HG excluded) | To assess the effects of follow-up counselling on the duration and severity of NVP | Demographic data  PROMs  PUQE (for recruitment) (PUQE version not specified),  NVPI (Nausea and vomiting in pregnancy instrument) (outcome measure) | Counselling effectively reduced the duration and severity of mild or moderate nausea and vomiting during pregnancy. However, it did not affect the duration of severe NVP | Full text |
| Professional support intervention including individualised health education and supportive phone calls. Individual health education was provided using the booklet Health Guidelines for Nausea and Vomiting During Pregnancy (developed by researchers) to provide 1. Professional information on strategies such as diet and lifestyle changes to relieve NVP symptoms) and a chance to clarify questions and hold discussions, and 2. Emotional support from professionals. Telephone follow up included greeting and listening, clarifying individual questions, providing information and evaluating recommended strategies  **Liu et al 2014 (48).** | Taiwan | Quasi-experimental (non-randomisedpre-test post-test design with control group).  NVP  Inclusion: 6-12 weeks gestation, singleton pregnancy, scored 3 or higher on the INVR. HG was excluded and use of medications other than B6. | To examine the effectiveness of a professional support (PS) intervention (including individualised health education and supportive phone calls) in reducing the severity of nausea and vomiting and improving the quality of life of women in early pregnancy. | Body weight, severity of nausea and vomiting, quality of life and perceived level of symptom distress.  Questionnaire to collect demographic characteristics, level of perceived stress and information about whether the women had been provided with information about symptom relief.  PROMs:  Rhodes Index of Nausea, Vomiting, and Retching (INVR).  NVPQoL  A Visual Analogue Scale (VAS) for symptom distress. | Severity of NVP and perceived level of symptom distress were significantly lower in the experimental group than in the control group during weeks 2 and 4, and the women in the experimental group showed a significant improvement in their QOL in week 4. Differences in body weight at week 4 were not significant. This intervention could be routinely applied in prenatal nursing health education. | Full text |

Mobile applications (n = 3 MoC from 5 records)

| **Description (ref)** | **Country** | **Study design and population** | **Aim** | **Outcome measures and data sources (incl. PROMs/PREMs)** | **Key findings** | **Type of publication and other notes/ limitations** |
| --- | --- | --- | --- | --- | --- | --- |
| Ministart mobile app. Uses PUQE-24 to categorise NVP severity daily to show fluctuations over time and compare this to average symptoms. Treatment advice is provided based on their score (e.g., dietary and lifestyle advice for mild symptoms, and referral to the doctor for moderate -severe symptoms).  **Nordeng; Ngo et al 2022 (49, 50).** | Norway | RCT  NVP | To evaluate the impact of the mobile app on NVP related symptoms, QoL and decisional conflict regarding NVP treatment compared to standard maternal care (control). | PUQE-24, NVPQoL, Decisional conflict scale (DCS), knowledge statements, reported sick leave and duration, hospitalisation duration, beliefs about medication and risk evaluation in questionnaires.  PROMs  PUQE-24, NVPQoL, Decisional conflict scale (DCS) | 268 pregnant women with NVP, enrolled at a median of 8 gestational weeks (range 4-36 weeks).  192 (86.5%) completed baseline questionnaires and were randomised into intervention (n=89) or control group (n=103). The app had no impact on NVP severity, QoL, or decisional conflict regarding NVP compared with standard care. | Trial registration  Full text  15% of women in the intervention group were beyond the first trimester.  Selecting specific time points in the day for evaluating NVP severity may help more accurately captures changes overtime.  25% drop out rate. Study did not reach target recruitment.  Findings may therefore have been influenced by gestation at enrolment, women’s parity, time to follow up and sample size. |
| iPhone Hyperemesis Gravidarum Care App. Developed to improve accuracy in defining symptom levels of HG, communication between providers and patients with HG, and treatment of HG. It functions to track medication, food, fluid, and vitamin intake; symptoms including nausea, vomiting, retching, urination, bowel movements and weight; and changes in NVP levels using the PUQE Score and also calculates the HELP score.  **Korouri et al 2019 (51).** | USA | Observational  Descriptive mixed methods  NVP and HG | To evaluate the success of the App in defining symptom levels, improving communication with healthcare providers, and improving overall quality of care as well as identifying areas for improvement. | Patient and healthcare provider feedback survey responses and App download and usage statistics after 7 days of use and review at their next prenatal appointment.  PROMs (as part of clinical care)  PUQE (version not specified)  HELP Score  PREMs (as part of evaluation)  Study specific questionnaire on experience of using the app. | 60/339 invited patients with severe NVP and HG used the app for 7 days, of which 36 (60%) completed the feedback survey. Patients felt positive about the app’s ability to accurately define symptoms levels (92%), improve communication (66%), and improve care (61%). Providers (n=6) unanimously thought the HG Care app was accurate in defining symptoms levels and was useful in improving communication, and most (67%) found it useful in improving care. | Full text  Participant demographic information was not collected. Changes in care based on the app was not evaluated, nor was the impact upon hospitalisation. |
| CheckWare software. Web-based data collection system that can collect information about NVP symptoms and quality of life from women to assess changes overtime and then generate advice that is sent via text message based on a predefined pathway.  **McParlin and Robson 2019; McParlin 2019 (52, 53).** | UK | Observational  NVP | To assess whether a web-based data collection system could be used to gather research data on three occasions over 7 days, facilitating patient-initiated responses. | Demographics, symptom severity (PUQE) and quality of life questionnaires were completed directly onto the website, with follow up questionnaires completed at 3 and again 7 days later (3 points total).  PROMs  PUQE (version not specified)  QoL questionnaire (not specified) | Of 208 women that consented to participate, 39.4% completed both follow up questionnaires, 24.5% completed one, and 36.1% completed none. Follow up completion was not related to symptom severity. 58% of participants who did complete PUQE scores had worsening symptoms over 7 days. Use of the web-based program to collect participant data was feasible and acceptable, however completion rates were disappointing. | Abstract  Website |

**Group-based care** (n = 8 MoC from 11 records)

| **Description (ref)** | **Country** | **Study design and population** | **Aim** | **Outcome measures and data sources (incl. PROMs/PREMs)** | **Key findings** | **Type of publication and other notes/ limitations** |
| --- | --- | --- | --- | --- | --- | --- |
| Small group teaching – presented by a researcher over 8 sessions (each 30-45 minutes) provided twice a week for 4 weeks compared to routine care. Three intervention groups each consisting of 10 members. Recommendations included: diet and lifestyle modification, methods for reducing fatigue, and improving mental status during pregnancy. Participants could also call the researcher for advice during the study period.  **Kamali et al 2018; Irct2016071328911N 2017 (54, 55).** | Iran | RCT  NVP (HG excluded)  Recruiting 59 women with mild-mod NVP between 6-12 weeks gestation. Exclusions included using anti-nausea medications in the past week. | To evaluate the effect of small group teaching on quality of life in pregnant women with nausea and vomiting. | PROMs  Modified PUQE (for recruitment)  NVPQoL (outcome measure) | There was a significant difference between the mean QoL score in the intervention and control group after the intervention (p=0.001). QoL score was significantly different before and after the intervention in the intervention group (p=0.001). | Full text  Trial registration |
| The intervention included two 60-minute group training sessions (provided 3 days apart) based on the Ottawa nutritional guideline compared with routine care. 3-5 cases were presented in a the form of lecture (40 min), questions and answers and group discussion (20 mins). Sessions covered: lifestyle changes, alternative medicine, acupressure, rest positions. Booklet were handed out. The intervention group received phone calls twice a week within 3 days for two weeks afterwards (week 3 and 4).  Golmakani et al 2017; **Irct2015011920716N (56, 57).** | Iran | RCT  (single blind)  NVP (HG excluded)  Recruiting 60 women between 6-11 weeks gestation not taking medication for NVP (Vit B6 ok). Score of 3-16 on the INVR. Low DASS scores. HG was excluded. | To determine the effect of an educational intervention (based on the Ottawa nutritional guideline) on health-related quality of life in women with NVP. | Subject selection form, demographic and midwifery data form, Rhodes index of nausea, vomiting and retching, health-related quality of life for NVP (NVPQoL) questionnaire, DASS-21, and checklist of Ottawa guideline implementation.  PROMs  NVPQoL (evaluation)  INVR (for recruitment)  DASS-21 (for recruitment) | The mean NVPQoL score was significantly different between the intervention and control group after the study (P<0.001), as was the difference between pre and post intervention scores in the intervention group (P<0.001). | Full text  Trial registration |
| The Ottawa nutritional guideline was presented in two sessions of 40 minutes each (3-5 cases) in the form of a lecture (20 minutes), questions, answers and group discussion (20 minutes) and compared to a control group. This guideline aims to provide evidence-based instructions for the early treatment of NVP and suggests strategies for early diagnosis and treatment of this condition. It includes recommendations about lifestyle modifications, nutritional therapies, complementary medicine and acupressure. There was two days between the two sessions. Arabic booklets were distributed to intervention group participants to take home in addition to samples of foods recommended.  **Toukhi et al 2023 (58).** | Egypt | RCT (single blind)  NVP (HG excluded)  60 pregnant women (28 in intervention and 27 in control group). Inclusion criteria: 7-13 weeks gestation, Singleton pregnanc, PUQE-24 score between 7-12, not receiving medication for NVP (except B6) (women with HG were excluded). | To examine the impact of applying the Ottawa nutritional guidelines on the control of NVP | Structured interview questionnaire (demographics, history of pregnancy and NVP), modified PUQE taken at 4 points each two days apart, also quality of life (data collection tool not specified).  PROMs  PUQE-24  QoL (tool not specified) | The severity and frequency of nausea and vomiting reduced significantly after starting the treatment compared to the control group. There was a significant difference between PUQE-24 score and QoL before and after the intervention for NVP. | Full text |
| Educational program involving two 60-minute sessions in groups of 2-5 people compared to a control group. Ottawa nutrition guide training (general guidelines, nutritional recommendations, other recommendations on lifestyle and acupressure) with lecture methods, question and answer and group discussion. The content is also provided in a booklet. Follow up phone calls are made to answer questions and reiterate information provided (twice a week three days apart for two weeks).  **Irct20180218038783N 2023 (59).** | Iran | RCT (single blind)  NVP (HG excluded)  60 (target sample size). Inclusion criteria: 4-16 weeks gestation, 15-45 years, not using drugs for NVP (except B6), score 3-16 in Rhodes questionnaire, BMI < 30. | To investigate the effect of an educational intervention based on the Ottawa Nutritional guide on NVP. | Primary outcome: Average total score of nausea, vomiting and belching in pregnant women during the first two weeks from the beginning of the study.  Secondary outcome: Average sexual satisfaction score before the beginning and after the fourth week of the intervention.  PROMs  INVR  Hudson sexual satisfaction questionnaire. | N/A | Trial registration |
| Six sessions of group counselling based on cognitive behavioural therapy compared to usual care.  **Irct20200530047606N 2020 (60).** | Iran | RCT  NVP  Recruiting  136 women with NVP and anxiety between 6-12 weeks gestation. | Not specified | PROMs: Anxiety (PRAQ-17) and NVP (PUQE – version not specified) | N/A | Trial registration |
| Counselling with hope therapy approach (8 group sessions held twice a week with each session lasting 90 minutes) compared with usual prenatal care and diet. Women will become familiar with concepts such as hope, growth of hope and will be encouraged to share their life stories. This will involve goal setting and strategies to achieve set goals including motivating pathways.  **Irct20150731023423N 2023 (61).** | Iran | RCT  NVP  68 (target sample size) women with NVP  6-12 weeks gestation, low risk pregnancy, specific scores on Rhodes nausea and vomiting questionnaire and Cohen’s perceived stress questionnaire. | To evaluate the effect of counselling with hope therapy approach on improving perceived stress and quality of life in women with NVP. | NVPQoL before and 8 weeks after the end of the intervention.  Cohen’s perceived stress questionnaire before and 8 weeks after the end of the intervention.  Secondary outcome – Hope for Life Scale before intervention and 8 weeks after the end of the intervention.  PROMs  Rhodes Nausea and Vomiting Questionnaire (recruitment)  Cohen’s Perceived Stress Questionnaire (recruitment and evaluation)  Edinburgh Questionnaire (recruitment)  NVPQoL (evaluation)  Hope for Life Scale (evaluation) | N/A | Trial registration |
| Group biofeedback (intervention group only), mental health education and usual treatment. A nurse who received professional biofeedback training will help participants to learn diaphragmatic breathing techniques, Jacobson’s muscle relaxation and guided Imagery, while monitoring heart rate variability. The intervention will include 10 sessions delivered every working day in 2 weeks (2-4 women per group each lasting 30-40 minutes). This is provided to inpatients and outpatients (if they are discharged prior to 2 weeks).  **Ciu et al 2022; ChiCtr 2020 (62, 63).** | China | RCT  HG  Target sample size is 68 women with HG diagnosed with at least one psychosomatic syndrome. | To examine the efficacy of group biofeedback treatment on patients with HG with psychosomatic symptoms, which will be evaluated through the revised version of Diagnostic Criteria for Psychosomatic Research (DCPR-R). | Psychosomatic syndrome, heart rate variability, severity of HG, QoL.  Hospital days, repeated treatment times, lab investigations.  Modified PUQE; HRV (heart rate variability) index; Short form Health survey (SF-12) for QoL.  PROMs  Modified PUQE; Short form Health survey (SF-12) for QoL. | N/A | Protocol  Trial registration |
| Two-week nursing care program (NCP), based on Miwa’s reflective practice, including 3 meeting style interventions, two phone calls and a self-recorded diary.  **Iwakuni 2017 (64).** | Japan | Quasi-experimental study (non-randomised)  NVP  Women with NVP. 45 (intervention) 67 (control) | To evaluate the effects on self-care agency (ESCA35) and NVP (INVR) by a two-week NCP aimed for pregnant women to improve self-care behaviour to find their own ways to alleviate NVP. | PROMs  self-care agency (ESCA35) and NVP (Rhodes Index of nausea, vomiting and retching) | The variations in subscale score of ESCA35 were larger in the intervention group. Three subscale scores of INVR (retching experience, retching occurrence, and total distress) were significantly improved only in the intervention group. Scores after 2 weeks showed no significant differences between the two groups. | Abstract |

**Primary care** (n = 6 MoC from 8 records)

| **Description (ref)** | **Country** | **Study design and population** | **Aim** | **Outcome measures and data sources (incl. PROMs/PREMs)** | **Key findings** | **Type of publication and other notes/ limitations** |
| --- | --- | --- | --- | --- | --- | --- |
| Standard Operating Procedures (SOPS) for General Practitioners. Two awareness sessions held three months apart by a gynaecologist and a paediatrician aimed at communicating consultant practice. Gynaecologist prepared standing procedures (SOPS) and tips for management of common problems in pregnancy.  **Azam et al 2016 (65).** | Pakistan | Quasi-experimental (pre-post test)  HG  10 Female GPs from two family welfare centres | To determine the effectiveness of educating doctors in improving the referral rate and quality in a public-sector healthcare setting. | Referral numbers to gynaecology for HG.  No PROMs or PREMs | In gynaecology there was an 31% reduction in HG referrals during the first trimester, reflecting improved standard of counselling. | Full text  Included outcomes other than HG referrals. Not clear what GPs were advised to assess to determine referral for HG. |
| Community pharmacy consultation either in person or over the phone involving 14 community pharmacies (15 pharmacists).  **Truong et al 2020; Truong et al 2020 (66, 67).** | Norway | RCT  NVP/HG (mod/severe NVP)  Pregnant women (some with NVP). 245 pregnant women (of which 114 had mod/severe NVP – 47.9% in intervention and 45.2% in control group) | To investigate the impact of a pharmacist consultation on pregnant women’s quality of life focusing on NVP and patient satisfaction. | Online questionnaires at baseline and during second trimester + satisfaction questionnaire for intervention group.  PROMs  Quality of Life Scale (QOLS)  PREMs  Satisfaction questionnaire. | Satisfaction with the consultation was high with most finding it useful and wanting it again in future.  Consultation did not have an overall impact on QOLS from baseline to 13 weeks post (second trimester), however secondary analysis showed the effect did differ according to NVP severity. | Full text  Abstract  This study did not exclusively recruit women with NVP. Future studies should further explore the effect of a pharmacist consultation specifically for NVP and on other outcomes such as use of health care services and medication use in pregnancy. |
| SafeStart study. Community pharmacy consultation. Intervention group: pharmacist consultation to address concerns and needs regarding medications and ailments in pregnancy. Control group: standard care.  **Truong et al 2019; Truong et al 2019 (68, 69).** | Norway | RCT  NVP | To test the feasibility of a pharmacist consultation in early pregnancy and to develop a patient centered mobile app for NVP. | PROMs  PUQE (baseline only) (PUQE-24)  Wellbeing score (0-10) (baseline only)  PREMs Patient satisfaction questionnaire (study specific) | Treatment of NVP and general information about medication were most frequently discussed during consultations and women reported high satisfaction. Having the option of telephone and follow up consults was important. | Abstract  Full text |
| The intervention involved three face-to-face counseling sessions based on the model of continuous midwife-led care. The control group received routine prenatal care. The intervention group received three face-to-face counselling sessions (45-60 minutes each).  **Moghtader et al 2022 (70).** | Iran | RCT  NVP (HG excluded)  The study included 60 pregnant women aged 8-12 weeks with mild to moderate NVP with no use of antinausea medication. HG was excluded. | To assess the effect of counselling based on the model of continuous midwife-led care on nausea and vomiting during pregnancy. | Severity and frequency of nausea and vomiting during pregnancy, assessed using the Pregnancy-Unique Quantification of Emesis (PUQE) questionnaire.  PROMs  PUQE (evaluation) (PUQE-24) | In the second week of the study, with the continuation of the follow-up process and an increase in the number of counseling sessions, the mean overall severity of nausea and vomiting, as well as the mean retching episodes, decreased significantly compared to the control group. | Abstract |
| Three individual face-to-face support counselling sessions with a midwife (once per week), and continuous follow-ups via phone or online. Also using a 24hr P6 acupressure wristband for 14 days Vs. the same sessions and a placebo wristband.  **Moghtader et al 2023 (71).** | Iran | RCT  NVP (HG excluded)  Inclusion criteria: 8-12 weeks gestation and mild to moderate NVP (PUQE-24 score 4-12), also no history of acupuncture or acupressure. HG was excluded. | The effect of supportive counselling and acupressure on P6 point on nausea and vomiting compared to counselling alone. | PUQE-24 (nausea and vomiting) for 17 days (3 days prior to study and 14 days of the study).  PROMs  PUQE-24 | There was a decreasing trend in the mean scores of questions 1, 2 and 3 after the intervention (p<0.001). But the trend of decreasing scores in the intervention groups was more than the control group, although there was no significant difference. | Full text |
| Development of an Urgent Care nursing care clinical pathway for NVP/HG. The proposed pathway offers nurse-led assessment and plan of action.  **Coman et al 2023 (72).** | New Zealand | 4-month retrospective quality audit  Observational  NVP and HG  All women presenting with pregnancy related complaints, including NVP and HG (n=88). | To:   - improve the timelines of service delivery to pregnant women presenting with NVP; - minimise the risk of potential complications associated with severe dehydration - contribute to the identification of NVP to enable effective management at an Urgent Care clinic. | Data collected for all patients who presented during a four month period in 2020-2021 including wait time, treatment time, and time until discharge from service.  No PROMs or PREMs | Maternity presentations represented 0.5% of total presentations, of which 60% were NVP/HG. 56% of NVP/HG presentation had waited longer than their target time to be assessed and have treatment initiated. A subset of patients visited the facility with NVP on multiple occasions, presenting between 3-9 times with NVP. | Full text |

**Table references**

1. Buchanan G, Hendry J, Currie H. Management of women with acute nausea and vomiting in pregnancy in Dumfries and Galloway. BJOG: An International Journal of Obstetrics and Gynaecology. 2016;123(Supplement 2):236.
2. Channing S, Doraiswamy J. Implementation of day case management of Hyperemesis Gravidarum. BJOG: An International Journal of Obstetrics and Gynaecology. 2021;128(SUPPL 1):28.
3. Cobb A, Donovan C, Hookway T, Thakar R. A Hydration unit for hyperemesis gravidarum: Audit and re-audit. BJOG: An International Journal of Obstetrics and Gynaecology. 2014;121(SUPPL. 6):66.
4. Fernandopulle N, Doraiswamy J. Day case pathway for patients with hyperemesis: A qualitative improvement project. BJOG: An International Journal of Obstetrics and Gynaecology. 2021;128(SUPPL 2):223-4.
5. Ijaz S, Hudders N, Yang P, Ewies A. Audit on ambulatory management of hyperemesis-a successful and cost effective service. BJOG: An International Journal of Obstetrics and Gynaecology. 2016;123(Supplement 2):75.
6. Khan T, Karpate S, Shehmar M. Hyperemesis day centre audit. BJOG. 2013;120:527-8.
7. Doherty J, McHale H, Killeen S-L, Curran S, Bennett M, Sheehy L, et al. Women's experiences of Hyperemesis Gravidarum (HG) and of attending a dedicated multi-disciplinary hydration clinic. Women and birth : journal of the Australian College of Midwives. 2023.
8. O'Brien EC, Doherty J, Killeen SL, Bennett M, Murtagh L, Curran S, et al. The IRIS clinic: A Protocol for a mixed-methods study evaluating the management of Hyperemesis Gravidarum. Contemporary Clinical Trials Communications. 2023.
9. Pilling M, Knowles C, Khalil H. Nausea and vomiting in pregnancy (NVP): Standardising local practice and introducing ambulatory care. BJOG: An International Journal of Obstetrics and Gynaecology. 2018;125(Supplement 3):65.
10. Tompsett H, Ajala T, Dixon G, Kelly T. The implementation of an ambulatory care bundle for the treatment of hyperemesis gravidarum. BJOG: An International Journal of Obstetrics and Gynaecology. 2013;120(SUPPL. 1):477.
11. Ucyigit MA. Outpatient management of hyperemesis gravidarum and the impact on inpatient admissions; A retrospective observational study. European journal of obstetrics, gynecology, and reproductive biology. 2020;254:298-301.
12. Wang SJ, Ma K. The ambulatory hyperemesis gravidarum initiative: A quality improvement project. BJOG: An International Journal of Obstetrics and Gynaecology. 2018;125(Supplement 3):37.
13. Ajufo I. Four-year retrospective review of day admission service provision for hyperemesis gravidarum (HG) at St Thomas' Hospital London [UK]. BJOG: An International Journal of Obstetrics and Gynaecology. 2013;120(SUPPL. 1):534-5.
14. Clark JIM. Title optimising care for short stay, ambulatory and emergency gynaecology in exeterauthormr jim clark, consultant. Bipolar Disorders. 2016;18(Supplement 1):275.
15. Coleman L, O'Sullivan M, Dilloway L, Sinha A, Epee M. An innovative ambulatory care service for women suffering with hyperemesis gravidarum. BJOG: An International Journal of Obstetrics and Gynaecology. 2014;121(SUPPL. 2):19.
16. Fletcher SJ, Waterman H, Nelson L, Carter LA, Chuang LH, Roberts C, et al. The effectiveness and cost-effectiveness of a holistic assessment and individualised package of care of women with hyperemesis gravidarum: Randomised controlled trial. BJOG: An International Journal of Obstetrics and Gynaecology. 2013;120(SUPPL. 1):552-3.
17. Fletcher SJ, Waterman H, Nelson L, Carter LA, Dwyer L, Roberts C, et al. Holistic assessment of women with hyperemesis gravidarum: A randomised controlled trial. International journal of nursing studies. 2015;52(11):1669-77.
18. Lloyd J, Ramskill N, Sharma B. Improving management of patients with hyperemesis. BMJ quality improvement reports. 2014;2(2).
19. Nam SM, Patil P, Sieunarine K. A quality improvement project: An audit of the new management protocol for nausea and vomiting in pregnancy, and hyperemesis gravidarum. BJOG: An International Journal of Obstetrics and Gynaecology. 2018;125(Supplement 3):70.
20. Laitinen L, Nurmi M, Kulovuori N, et al. Usability of Pregnancy-Unique Quantification of Emesis questionnaire in women hospitalised for hyperemesis gravidarum: a prospective cohort study. BMJ Open. 2022;12(5).
21. Mitchell-Jones N, Farren J, Tobias A, Bobdiwala S, Bourne T, Bottomley C. Efficacy of outpatient management of severe nausea and vomiting (hyperemesis gravidarum): A randomised controlled trial and patient preference trial. BJOG: An International Journal of Obstetrics and Gynaecology. 2017;124(Supplement 1):7.
22. Mitchell-Jones N, Farren JA, Tobias A, Bourne T, Bottomley C. Ambulatory versus inpatient management of severe nausea and vomiting of pregnancy: a randomised control trial with patient preference arm. BMJ open. 2017;7(12):e017566.
23. Isrctn. Hyperemesis In Pregnancy (HIP) Trial: inpatient versus outpatient management of severe nausea and vomiting in pregnancy. https://trialsearchwhoint/Trial2aspx?TrialID=ISRCTN24659467. 2014.
24. McParlin C, Carrick-Sen D, Steen IN, et al. Hyperemesis in Pregnancy Study: a pilot randomised controlled trial of midwife-led outpatient care. European Journal of Obstetrics & Gynecology and Reproductive Biology. 2016;200:6-10.
25. McCarthy FP, Murphy A, Khashan AS, et al. Day care compared with inpatient management of nausea and vomiting of pregnancy: A randomized controlled trial. Obstetrics & Gynecology. 2014;124(4):743-8.
26. Murphy A, McCarthy FP, McElroy B, et al. Day care versus inpatient management of nausea and vomiting of pregnancy: cost utility analysis of a randomised controlled trial. European Journal of Obstetrics & Gynecology and Reproductive Biology. 2016;197:78-82.
27. Dean C, Marsden J. Satisfaction for treatment of hyperemesis gravidarum in day case settings compared to hospital admissions. MIDIRS Midwifery Digest. 2017;27(1):11-20.
28. Dean C, Marsden J. Women's experiences of treatment for hyperemesis gravidarum in day case settings compared to hospital admissions. MIDIRS Midwifery Digest. 2017;27(2):177-86.
29. Ryan N, Zhou C, Sewell T, et al. The Case for Day Case: Reduced Inpatient Stays with the use of Day Case Management in Hyperemesis Study. Current Women's Health Reviews. 2019;15(2):130-6.
30. Hordern CE, Medina Lucena H, Stanley KP, Sule MM. HARP-Hyperemesis Ambulatory Rehydration Project: Development, implementation and improvement to minimise hospital stay. BJOG: An International Journal of Obstetrics and Gynaecology. 2013;120(SUPPL. 1):432-3.
31. Canty MA, Alexandrou E, Dickson H, Lawson K, Schmeid V, van Vorst MD, et al. Intravenous fluids for Moderate to Severe nausea and vomiting in pregnancy In the Community (IMSIC): Implementation protocol. Women and Birth. 2022;35(Supplement 1):44.
32. Carlon MN, Kiroglu MS. MOaH- Women, Midwives and Doctors working together in complex care. Women and Birth. 2022;35(Supplement 1):50-1.
33. Ostenfeld A, Futtrup TB, Lokkegaard ECL, Westergaard HB. Reorganising and improving quality of care for hyperemesis gravidarum in a Danish hospital: a quality improvement project. BMJ open quality. 2023;12(3).
34. Skalley G, Denny J, Allen E, Rao S. Optimisation of hyperemesis gravidarum management through an emergency department setting. BMJ open quality. 2018;7(3):e000330.
35. Chua-Gocheco A, Tan M, Kim E, Erebara A, Maltepe C, Koren G, Bozzo P. Summary of motherisk calls for 2011. Birth Defects Research Part A - Clinical and Molecular Teratology. 2012;94(5):408.
36. Madjunkova S, Maltepe C, Koren G. The leading concerns of American women with nausea and vomiting of pregnancy (NVP) calling motherisk NVP helpline. Birth Defects Research Part A - Clinical and Molecular Teratology. 2013;97(5):367.
37. Madjunkova S, Maltepe C, Koren G. The Leading Concerns of American Women with Nausea and Vomiting of Pregnancy Calling Motherisk NVP Helpline. Obstetrics and gynecology international. 2013;2013:752980.
38. Madjunkova S, Maltepe C, Farine D, Koren G. Patterns of antiemetic use among american women with nausea and vomiting of pregnancy. Obstetrics and Gynecology. 2014;123(SUPPL. 1):155S.
39. Maltepe C, Vlad Popa M, Bertucci C, Farine D, Koren G, Nulman I. The effects of counseling and predictors of pregnancy outcomes in women with hyperemesis gravidarum. Obstetrics and Gynecology. 2015;125(SUPPL. 1):101S.
40. Godbole KG, Kulkarni SS, Godbole GP, Kulkarni AM. Experiences from Garbha-Swasthya helpline. Indian journal of public health. 2015;59(2):149-52.
41. Patil AS, Patil NP, Lewis AN, Swamy GK, Murtha AP. Health care providers' use of a drug information service for pregnancy-related inquiries. Journal of the American Pharmacists Association. 2014;54(5):502-9.
42. Heitmann K, Bakkebo T, Havnen GC, Schjott J. Breastfeeding women in need of information about antiemetics for nausea and vomiting during pregnancy: a review of inquiries to a medicines information service. Frontiers in pharmacology. 2023;14:1238875.
43. Singh A, Agarwal A, Thakur G, Goel S, Singh M. Role of telemedicine in obstetrics and gynecology: an experience at tertiary care center. Expert Review of Medical Devices. 2023;20(12):1251-6.
44. Abedian Z, Abbaszadeh N, Latifnejad Roudsari R, Shakeri MT. The effect of telephone support on the severity of nausea and vomiting in the first trimester of pregnancy in the primiparous women. Iranian Journal of Obstetrics, Gynecology and Infertility. 2014;17(118):22-9.
45. Irct2014012316326N. The effect of telephone support on health-related quality of life in nausea and vomiting of pregnancy. https://trialsearchwhoint/Trial2aspx?TrialID=IRCT2014012316326N1. 2014.
46. Abedian Z, Abbaszadeh N, Roudsari RL, Shakeri MT. The effects of telephone support on stress and perceived social support in primiparous women experiencing nausea and vomiting in the first half of pregnancy. Journal of Midwifery & Reproductive Health. 2015;3(2):328-34.
47. Isbir GG, Mete S. The effect of counselling on nausea and vomiting in pregnancy in Turkey. Sexual & reproductive healthcare : official journal of the Swedish Association of Midwives. 2016;7:38-45.
48. Liu MC, Kuo SH, Lin CP, Yang YM, Chou FH, Yang YH. Effects of professional support on nausea, vomiting, and quality of life during early pregnancy. Biol Res Nurs. 2014;16(4):378-86.
49. Nordeng, H. MinSafeStart - Decision Aid Tool for Better Treatment of Nausea and Vomiting During Pregnancy. [https://clinicaltrialsgov/show/NCT04719286. 2021](https://clinicaltrialsgov/show/NCT04719286.%202021).
50. Ngo E, Truong MB, Wright D, Nordeng H. Impact of a Mobile Application for Tracking Nausea and Vomiting During Pregnancy (NVP) on NVP Symptoms, Quality of Life, and Decisional Conflict Regarding NVP Treatments: minSafeStart Randomized Controlled Trial. JMIR mHealth and uHealth. 2022;10(7):e36226.
51. Korouri E, MacGibbon K, Chan M, Guba L, Dela Cruz L, Leung JW, et al. Performance of iPhone Hyperemsis Gravidarum Care App. Journal of clinical case reports and case studies. 2019;2(1).
52. McParlin C, Robson S. INPUT: Impact of nausea on pregnancy using technology: Feasibility of monitoring the trajectory of symptom severity in women suffering from nausea and vomiting in pregnancy using 'CheckWare' software. BJOG: An International Journal of Obstetrics and Gynaecology. 2019;126(Supplement 1):52.
53. McParlin C. Acceptable, easy to use, convenient and less burdensome! : EG CheckWare; 2018 [Available from: <https://www.checkware.com/post/acceptable-easy-to-use-convenient-and-less-burdensome>.
54. Kamali Z, Abedian Z, SaberMohammad A, Dehnavi ZM. The effect of small group teaching on quality of life in pregnant women with nausea and vomiting: A clinical trial. Journal of education and health promotion. 2018;7:112.
55. Irct2016071328911N. Effect of educational program on quality of life and health literacy. https://trialsearchwhoint/Trial2aspx?TrialID=IRCT2016071328911N1. 2017.
56. Golmakani N, Soltani M, Mobarhan MG, Mazloum SR. Evaluation of the Effects of an Educational Intervention Based on the Ottawa Nutritional Guideline on Health-Related Quality of Life in Pregnant Women with Nausea and Vomiting. Journal of Midwifery & Reproductive Health. 2017;5(2):873-81.
57. Irct2015011920716N. The effect of educational intervention based on Ottawa nutritional guidelines on nausea and vomiting and health-related quality of life in women. https://trialsearchwhoint/Trial2aspx?TrialID=IRCT2015011920716N1. 2015.
58. Toukhi NME, El Sayed YA, Zaghloul AS, Assar MM. Effect of Implementing Ottawa Nutritional Guidelines on the Course of Nausea and Vomiting During Pregnancy (A Randomized Control Trial). NeuroQuantology. 2023;21(5):1232-47.
59. Irct20180218038783N. Investigating the ?Effect of Educational Intervention based on the Ottawa Nutritional guide on Nausea and Vomiting in Pregnant women. https://trialsearchwhoint/Trial2aspx?TrialID=IRCT20180218038783N5. 2023.
60. Irct20200530047606N. The effect of group counseling based on cognitive-behavioral therapy onanxiety and nausea and vomiting in pregnant women. https://trialsearchwhoint/Trial2aspx?TrialID=IRCT20200530047606N1. 2020.
61. Irct20150731023423N. The effect of counseling with hope therapy approach on improving perceived stress and quality of life of women suffering from nausea and vomiting of pregnancy. https://trialsearchwhoint/Trial2aspx?TrialID=IRCT20150731023423N22. 2023.
62. Cui X, Cao J, Rafanelli C, Zhu B, Gostoli S. Efficacy of group biofeedback treatment on hyperemesis gravidarum with psychosomatic symptoms diagnosed with the revised version of Diagnostic Criteria for Psychosomatic Research (DCPR-R): study protocol for a randomised controlled trial. BMJ open. 2022;12(3):e051295.
63. ChiCtr. Group Biofeedback Treatment for Hyperemesis Gravidarum with Psychosomatic Symptoms: a Randomized Controlled Trial. https://trialsearchwhoint/Trial2aspx?TrialID=ChiCTR2000028754. 2020.
64. Iwakuni A. Verification of the effectiveness of a nursing support program aimed at improving self-care abilities and alleviating morning sickness through the exploration of self-care actions suitable for pregnant women. Journal of japan academy of nursing science. 2017;37(1):353‐63.
65. Azam N, Hassan U, Farooq A. Effect of continued professional development on clinical performance in a public sector health care setting. Journal of the Pakistan Medical Association. 2016;66(2):174-8.
66. Truong MB-T, Ngo E, Ariansen H, Tsuyuki RT, Nordeng H. The effect of a pharmacist consultation on pregnant women's quality of life with a special focus on nausea and vomiting: an intervention study. BMC pregnancy and childbirth. 2020;20(1):766.
67. Truong MBT, Ngo E, Ariansen H, Tsuyuki RT, Nordeng H. The effect of a pharmacist consultation in early pregnancy on pregnant women's quality of life: An intervention study. Pharmacoepidemiology and Drug Safety. 2020;29(SUPPL 3):541-2.
68. Truong MB, Ngo E, Ariansen H, Tsuyuki RT, Nordeng H. Community pharmacist counselling in early pregnancy - Results from the SafeStart feasibility study. PLOS One. 2019;14(7).
69. Truong MBT, Ngo E, Ariansen H, Nordeng H. Pharmacist counseling in early pregnancy and the development of a patient-centered mobile application for NVP: The safestart study. Birth Defects Research. 2019;111(9):553.
70. Moghtader AA, Masoumi SZ, Khodakarami B, Roshanaei G, Parsa P. Effect of Counseling Based on the Model of Continuous Midwife-led Care on Nausea and Vomiting During Pregnancy in Patients Referred to Comprehensive Health Centers in Hamadan, 2020. Avicenna Journal of Nursing and Midwifery Care. 2022;30(3):200-10.
71. Moghtader AA, Masoumi SZ, Khodakarami B, Roshanaei G, Parsa P. The effect of supportive counseling with acupressure on nausea and vomiting in pregnant women referring to ehalth centers in Hamadan, Iran: A randomised double blind controlled trial study. Journal of Postgraduate Medical Institute. 2023;37(1):3-11.
72. Coman RM, Richardson SK. Managing nausea and vomiting in pregnancy: development of a clinical pathway proposal in an urgent care setting. Emergency Nurse New Zealand. 2023;23(3):27-33.
